# Biomimetic Microfibers for Myelin-Enhancer Screening and Neural Regeneration

**DOI:** 10.34133/cbsystems.0565

**Published:** 2026-05-07

**Authors:** Lili Quan, Akiko Uyeda, Atsushi Sekiguchi, Ze Zhang, Kazuhisa Sakai, Tsunehiko Takamura, Ruijuan Zhang, Noritaka Ichinohe, Shinjiro Umezu, Rieko Muramatsu

**Affiliations:** ^1^ Department of Molecular Pharmacology, National Institute of Neuroscience, National Center of Neurology and Psychiatry, Tokyo 187-8502, Japan.; ^2^ Department of Psychosomatic Medicine, Graduate School of Medical Sciences, Fukuoka 812-8582, Japan.; ^3^ Department of Modern Mechanical Engineering, Graduate School of Creative Science and Engineering, Waseda University, Tokyo 169-8555, Japan.; ^4^ Department of Ultrastructural Research, National Institute of Neuroscience, National Center of Neurology and Psychiatry, Tokyo 187-8502, Japan.; ^5^ Department of Behavioral Medicine, National Institute of Mental Health, National Center of Neurology and Psychiatry, Tokyo 187-8502, Japan.; ^6^ Institute for Healthcare Robotics, Future Robotics Organization, Graduate School of Creative Science and Engineering, Waseda University, Tokyo 169-8555, Japan.

## Abstract

Remyelination requires the precise wrapping of axons by oligodendrocyte processes, a critical step for restoring neural circuit function. However, a lack of quantitative systems that recapitulate axonal geometry and chemistry has limited mechanistic and pharmacological insights into myelin wrapping. Here, we present a bioengineered microfiber platform that mimics neurite architecture and surface chemistry, enabling high-content quantification of oligodendrocyte wrapping. Through compound screening, we identified dimemorfan, a clinically used sigma-1 receptor agonist, as a potent enhancer of myelin wrapping. Dimemorfan treatment accelerated remyelination and functional recovery in demyelinated mice and promoted myelin wrapping by human induced pluripotent stem cell (iPSC)-derived oligodendrocytes. Moreover, population-level magnetic resonance imaging (MRI) analyses revealed increased white matter volume in dimemorfan-administered individuals. This study establishes a biomimetic materials platform for myelin quantification and regeneration-oriented drug discovery, providing mechanistic and translational insights into sigma-1 receptor-mediated control of myelin wrapping.

## Introduction

Myelin is a lipid-rich structure that ensheathes neuronal axons and plays an essential role in maintaining the integrity of neural circuits by facilitating saltatory conduction and supporting axonal metabolism [1,2]. Demyelination in the central nervous system (CNS) is a common pathological hallmark across various neurological diseases, such as multiple sclerosis (MS) [3], Alzheimer’s disease [4,5], and amyotrophic lateral sclerosis (ALS) [6]. Although demyelination is strongly associated with loss of neural function, there are currently no approved therapies that promote myelin repair, making it critical to elucidate the underlying mechanisms of remyelination [1]. Myelin is formed by a type of glial cell called oligodendrocytes and their precursor cells, oligodendrocyte precursor cells (OPCs). OPCs are widely distributed throughout the CNS across the lifespan, meaning that they are an attractive therapeutic target for many neurological disorders in which aging is a major risk factor [7]. Remyelination requires the proliferation and differentiation of OPCs, followed by the wrapping of axons [8]. Although OPCs remain abundant and proliferative even in the aged CNS [9], accumulating evidence suggests that the steps beyond differentiation, particularly axonal wrapping, are the limiting stages for functional remyelination [10].

A variety of in vitro platforms have been developed to dissect the cellular and molecular mechanisms underlying remyelination and to facilitate the identification of therapeutic compounds. In addition to coculture systems of neurons and oligodendrocytes for assessing myelination, methods have also been developed to evaluate oligodendrocyte wrapping on engineered microstructures, because oligodendrocytes are capable of wrapping even fixed neurons [11]. These systems, such as microfibers with axon-mimetic diameters [12,13] and micropillar arrays enabling high-throughput analysis [14], have proven effective in screening for promyelinating agents; for example, clemastine identified via engineering approaches has advanced to clinical trials in MS (NCT02040298). To further enhance the precision of outcomes obtained through such engineering approaches, a promising direction would be to establish an evaluation system that more closely mimics neuronal axons. In particular, incorporating axonal characteristics such as surface roughness and chemical cues that reflect the molecular expression profile of axons would address key processes required for effective remyelination.

In this study, we developed neurite-mimicking nanofibers to evaluate oligodendrocyte wrapping. We established a device-based assay system that enables quantitative assessment of oligodendrocyte wrapping alongside parallel measurements of OPC proliferation and differentiation, key processes required for promoting remyelination. Building on this, we show that activation of the sigma-1 receptor, which is the known mechanism of dimemorfan [15], drives axonal wrapping, and that compounds emerging from this platform not only promote remyelination in disease-model mice but also associate with improved myelin-related signals on human brain imaging after dimemorfan intake. These results highlight the potential of this platform as a translational screening system for identifying remyelination-promoting therapeutics.

## Materials and Methods

### Animals

Sprague–Dawley rats and C57BL/6J mice were purchased from Tokyo Laboratory Animal Science and Japan SLC. Mice were maintained at 22 °C with a 12-h light–dark cycle under specific pathogen-free conditions and were given ad libitum access to food and water. All experimental procedures were approved by the Committee on the Ethics of Animal Experiments of the National Institutes of Neuroscience, National Center of Neurology and Psychiatry (no. 2024035R1, 2024030).

### Fabrication of microfiber

The polycaprolactone (PCL) electrospinning solution was loaded into a syringe equipped with a nozzle in the electrospinning setup. The positive electrode of the power source was attached to the nozzle tip; the negative electrode of the power source was connected to a conductive stage covered with micro-cover glass (22 mm × 32 mm, C022321, Matsunami). To ensure uniformity and regularity of the PCL microfibers, near-field electrospinning was conducted under controlled environmental conditions of 25.4 °C and 53.7% humidity. Following electrospinning, fiber surface modification was performed by oxygen plasma etching at different radio frequency (RF) discharge power. Among the tested conditions, 75 W for control (to increase hydrophilicity of the fiber) and 200 W for etching condition under a reactor internal pressure of 20 Pa was selected as the standard setting. The 3 × 10 s condition was selected as the optimized plasma etching protocol because it provided sufficient surface activation for subsequent oligodendrocyte adhesion assays while preserving microfiber morphology. The resulting fibers had a diameter of approximately 2 to 5 μm. The minimum interfiber spacing was determined based on the reported size of mature oligodendrocytes (MOLs) in culture, which extend membrane sheets exceeding 50 μm in diameter [16,17]. The resulting microfiber morphology was characterized using a 3D Surface Profiler (VK-X3000 Series, Keyence), with surface roughness quantified by the arithmetic mean roughness (Ra) and the maximum height of the profile (Rz). Unless otherwise specified, the control fibers used in experiments shown here 2 were subjected to plasma etching under these conditions.

### Scanning electron microscopy

Fiber samples were cut into small pieces and mounted onto aluminum stubs using conductive carbon tape. To eliminate charging effects arising from the low electrical conductivity of the fibers during observation and to enhance image resolution and surface detail contrast, a thin platinum layer was sputter-coated onto the samples prior to imaging. Sputter coating was performed under an argon atmosphere at room temperature, with a coating time of approximately 60 s, resulting in a coating thickness of about 5 nm. The surface morphology of the fibers was examined using a field-emission scanning electron microscope (S4800, Hitachi). Scanning electron microscopy (SEM) observations were carried out at an accelerating voltage of 25 kV, and images were acquired in secondary electron mode to evaluate the surface features and overall morphology of the fibers.

### Preparation of O4-positive oligodendrocytes

Cultures were prepared from P7 rat brain cortices. The cerebral cortices were dissected and minced with fine scissors in ice-cold phosphate-buffered saline (PBS). The minced tissues were dissociated with 0.25% trypsin–EDTA (Sigma) in PBS at 37 °C for 15 min. After neutralization with Dulbecco’s modified Eagle’s medium (DMEM; 12800082, Thermo Fisher Scientific) containing 10% fetal bovine serum (FBS) (F7524, Sigma-Aldrich), cells were centrifuged at 500*g* for 10 min. Cell pellets were suspended in 10% FBS–DMEM and then filtered through a 70-μm nylon cell strainer. Cells were plated on poly-l-lysine (PLL; P2636, Sigma-Aldrich)-precoated T-75 cell culture flasks at a density of 2.5 × 10^7^ cells/flask and maintained at 37 °C with 5% CO_2_ in 10% FBS–DMEM. Ten days after culturing, cultures were washed with PBS, and the remaining cells were treated with 0.05% trypsin–PBS. The detached cells were filtered through a 40-μm nylon cell strainer, followed by the isolation of O4-positive oligodendrocytes using anti-O4 microbeads (130-094-543, Miltenyi Biotec). Isolated cells were plated at 4,000 cells/well (384-well for differentiation assay) and 1 × 10^5^ cells/platform (for wrapping assay), maintained in myelin medium as previously described [13], consisting of DMEM supplemented with B27, N2, penicillin–streptomycin, N-acetylcysteine, and forskolin, with additional platelet-derived growth factor-AA (PDGF-AA, PeproTech). The detailed information was listed in Table S1. For the induction of oligodendrocyte differentiation, PDGF-AA will be removed from the myelin medium. To assess the effect of extracellular matrix (ECM) on wrapping, microfibers were precoated with the following reagents: PLL (0.1 μg/μl, P2636, Sigma), fibronectin (1 μg/μl, 354008, Corning), type IV collagen (0.023 μg/μl, 354245, Corning), and laminin fragment iMatrix-511 (0.1 μg/μl, 892012, Nippi). For in vitro screening, rat O4-positive oligodendrocytes or induced pluripotent stem cell (iPSC)-derived oligodendrocytes were plated onto a laminin fragment iMatrix-511-coated microfiber platform. Each coating was conducted at 4 °C overnight or 37 °C for 1 h prior to cell seeding.

### Immunocytochemistry analysis

After culturing, cells were fixed with 4% paraformaldehyde (PFA) in 0.1 M phosphate buffer (PB) for 30 min at room temperature. Samples were treated with blocking solution (0.1% Triton X-100 in PB), followed by incubation with 3% normal donkey serum (NDS) in PBS for 1 h at room temperature. Samples were incubated with the following primary antibodies diluted in the blocking solution overnight at 4 °C: rabbit anti-myelin basic protein antibody (MBP; 1:500, ab40390, Abcam), mouse anti-MBP antibody (1:500, MAB42278, R&D Systems), and goat anti-Olig2 antibody (1:300, AF2418, R&D Systems). Primary antibodies were detected by the following secondary antibodies diluted in PBS supplemented with 3% NDS and incubated for 1 h at room temperature: Alexa Fluor 488-conjugated donkey antibody against rabbit or mouse immunoglobulin G (IgG) (1:500, A21206 or A21202, Thermo Fisher Scientific) and Alexa Fluor 568-conjugated donkey antibody against goat IgG (1:500, A11057, Thermo Fisher Scientific). Images were captured using IN Cell Analyze 2000 (GE Healthcare) or a confocal laser scanning microscope (FV3000, Olympus), and were analyzed using ImageJ software [Fiji, version 2.16.0, National Institutes of Health (NIH)]. For quantification of the wrapping ratio, the proportion of MBP-positive fiber length relative to the total fiber length within the acquired images was calculated. MBP-positive fibers with lengths of 20 μm or more were defined as wrapped areas.

### MO3.13 cell culture

Human oligodendrocytic MO3.13 cell line (Cellutions Biosystems Inc.) was cultured in DMEM (12800-017, Thermo Fisher Scientific) supplemented with 10% FBS (F7524, Sigma), 100 μg/ml streptomycin, and 100 U/ml penicillin. Cells were passaged using 0.25% trypsin–EDTA (Sigma)–PBS and maintained at 37 °C with 5% CO_2_.

### Cell Counting Kit-8 assay

MO3.13 cells were plated in 384-well plates at 2,000 cells/well in the culture medium and were cultured with each compound at 2 μM for 24 h. Cell Counting Kit-8 (CCK-8, CK-04, Dojindo) reagent was added to the culture and incubated at 37 °C for 1 h. The absorbance was measured at 450 nm with a reference wavelength of 650 nm, using a microplate reader (SpectraMax 5, Molecular Devices).

### Human iPSC-derived oligodendrocytes

Human iPSC (hiPSC) (HPS0063, RIKEN Bioresource Center) were used for the preparation of human oligodendrocyte culture [18]. Cells were cultured in mTeSR1 medium with Y27632 (030-24021, Fuji film) and plated on Matrigel (354277, Corning)-precoated 6-well plate at a density of 1.5 × 10^5^ to 2 × 10^5^ cells. After 24 h, the culture medium was replaced with mTeSR1 without Y27632, and the cells were maintained in mTeSR1 until the colonies reached approximately 250 μm in diameter, followed by transition to neuronal induction medium to initiate differentiation toward PAX6^+^ neural stem cells, and the time point was defined as day 0. After 8 d of culture, cells were cultured in N2 medium for 4 d. On day 12, adherent cells were detached and transferred to low-attachment plates to promote the formation of Olig2-enriched aggregates, cultured in N2B27 medium to form spheres until day 20, and further cultured in PDGF medium for 10 d. From day 30, 300- to 800-μm spheres with well-defined morphology were selected and transferred onto poly-l-ornithine (P3655, Sigma-Aldrich)/laminin (23017015, Gibco)-coated dishes for adherent culture. Approximately 20 to 30 spheres were plated per well and maintained in PDGF medium, supporting OPC expansion. The cells are maintained with careful media changes, allowing the emergence of O4^+^ oligodendrocytes by day 75. For further maturation into myelinating oligodendrocytes, the culture medium is switched to a glial medium after day 75. Detailed compositions of each medium and reagent used are listed in Table S1.

### Lysophosphatidylcholine-induced demyelination model

Eight-week-old mice were anesthetized with isoflurane and then underwent a laminectomy at the Th11-12 vertebral level, followed by injection of 2 μl of 1% (*w*/*v*) lysophosphatidylcholine (LPC) (L1381, Sigma) into the central portion of the dorsal spinal cord at a depth of 0.5 mm. Mice received daily intraperitoneal administration of either dimemorfan phosphate (20 mg/kg per day, HY-B2215, Med Chem Express) or a vehicle control (filtered PBS) from 3 d after LPC injection until day 14. After surgery, the mice were randomly numbered and assessed in a blinded manner in the experimental groups for the ladder walk test.

### Experimental autoimmune encephalomyelitis

Eight-week-old mice were subcutaneously immunized with MOG_35–55_ (MEVGWYRSPFSRVVHLYRNGK, 100 μg, Sigma-Aldrich) in complete Freund’s adjuvant emulsion (CFA; Difco Laboratories) containing 500 μg of *Mycobacterium tuberculosis* H37Ra (Difco). Pertussis toxin (PTX; 200 ng, List Biological Laboratories) was intravenously administered immediately after immunization and again on day 2 [19]. Dimemorfan (20 mg/kg, HY-B2215, Med Chem Express) or a vehicle control (filtered PBS) was intraperitoneally injected daily to mice from 14 d after experimental autoimmune encephalomyelitis (EAE) induction. Clinical signs of EAE were scored on a 0 to 5 scale: 0, no signs; 0.5, tail tip droop; 1.0, partially tail droop; 1.5, tail paralysis; 2.0, hindlimb weakness; 2.5, one hindlimb paralysis; 3.0, both hindlimb paralysis; 3.5, hindlimb paralysis and forelimb weakness; 4.0, hindlimb paralysis and one forelimb paralysis; 4.5, hindlimb paralysis and both forelimb paralysis; 5.0, moribund or death. Spinal cords were collected at day 28 after EAE induction for histological analyses.

### Histology

Mice were transcardially perfused with PBS and 4% PFA in 0.1 M PB. The isolated samples were postfixed with 4% PFA in PB overnight at 4 °C and transferred to 30% sucrose in PBS overnight. Tissues were embedded in optimal cutting temperature (O.C.T.) compound (Tissue-Tek), frozen, and sectioned at 25 μm with a cryostat and then mounted onto Matsunami adhesive silane-coated glass slides. Myelin was stained using Black Gold II (Histo-Chem Inc.). Spinal cord sections were prepared with at least 6 slices that were 150 μm apart. Images were acquired using an inverted microscope (IX71, Olympus) and photographed by a digital microscope camera (DP80, Olympus). Quantification was performed using Fiji/ImageJ software (NIH).

### Electron microscopy

Mice were transcardially perfused with 2% glutaraldehyde and 2% PFA in 0.1 M cacodylate buffer (pH 7.2), and then the brains were postfixed with the same fixative overnight at 4 °C. Spinal cords were sectioned at 200-μm thickness using a Vibrating Blade Microtome (Leica VT1000) and then treated with 1% osmium tetroxide in cacodylate buffer for 60 min at room temperature. Samples were stained with 2% uranyl acetate for 60 min, serially dehydrated, and embedded in Epon821 (TAAB). Ultrathin sections (70-nm thickness) were cut using an ultramicrotome (EM UC6, Leica), and samples were observed with a Tecnai Spirit electron microscope (Thermo Fisher Scientific, FEI). The *g* ratio was quantified from >600 axons per animal. At least 6 fields of view per animal were used for the analysis. Calculations were performed using Fuji/ImageJ.

### Ladder walk test

Motor function in mice was assessed using a 1-m horizontal ladder test [20]. Prior to LPC injection, mice were habituated and trained to walk on a horizontal ladder equipped with stainless steel rungs arranged in an irregular spacing pattern, which was altered in each training session. Movements were recorded using a video camera, and the recordings were analyzed for the number of fault steps, including deep slips/misses, minor slips, and hind paw misplacements. The percentage of fault steps relative to the total number of steps was calculated to quantify motor performance.

### RNA-seq analysis

Total RNA was isolated from rat primary oligodendrocytes or iPSC-derived oligodendrocytes (OLs) using RNeasy mini kits (Qiagen). Full-length cDNA was synthesized from total RNA using the SMART-Seq HT Kit (Takara Bio, Japan). Illumina sequencing libraries were then prepared using the Nextera XT DNA Library Preparation Kit (Illumina). Sequencing was performed on the NovaSeq 6000 platform in a 101-base, single-end mode for rat samples and the DNBSEQ-G400 sequencer (MGI) in a single-end mode [150 base pairs (bp)] for human samples. The generated reads were mapped to the rat reference genome (Rn6) or human reference genome (hg19) using TopHat (version 2.1.1) after adapter trimming by Trimmomatic (version 0.38). Differential gene expression analysis was performed using DESeq2 (1.48.2) and Bioconductor packages in R (4.5.1). The RNA-seq data presented were derived from replicate 3 (GSE309050), and consistent trends were observed across all replicates (*n* = 3). Differentially expressed genes (DEGs) were defined by a fold change > 1.2 and an adjusted *P* value < 0.05 for rat samples, and a fold change > 1.5 and an adjusted *P* value < 0.1 for human samples.

For heatmap analysis, DEG expression values were normalized using *z* scores based on transcripts per million (TPM), and visualization was performed using the “pheatmap” R package in Bioconductor. Gene ontology (GO) analysis was conducted using the Database for Annotation, Visualization, and Integrated Discovery (DAVID, v2024q4) [21,22] or Metascape [23]. Gene set enrichment analysis (GSEA) was carried out using the “clusterProfiler” package in R (4.5.1), based on rank files generated from DESeq2-analyzed expression data. DEGs were analyzed using Ingenuity Pathway Analysis (IPA; QIAGEN) to explore representative signaling pathways.

### siRNA transfection

Human *SIGMAR1* small interfering RNA (siRNA) (A-017475-14) and nontargeting siRNA #1 (D-001910-01-05) were purchased from Dharmacon. Cells were seeded in 96-well plates at a density of 1 × 10^5^ cells per well and transfected with siRNA using Accell Delivery Media (B-005000, Dharmacon) at a final concentration of 1 μM per well. Knockdown efficiency of human *SIGMAR1* was assessed 3 d after siRNA transfection.

### Quantitative polymerase chain reaction

Total RNAs from the culture were extracted by TRIzol reagent (Thermo Fisher Scientific) and purified by RNeasy Micro Kit (Qiagen). For reverse transcription, cDNA was synthesized using the PrimeScript RT Master Mix (Takara Bio). Quantitative polymerase chain reaction (PCR) was performed using KAPA SYBR Fast Master Mix (KAPA Biosystems) with the following primer pairs: *SIGMA1R* forward, GTCCGAGTATGTGCTGCTCTTC; *SIGMA1R* reverse, GAAGACCTCACTTTTGGTGGTGC; *GAPDH* forward, AGGGCTGCTTTTAACTCTGGT; *GAPDH* reverse, CCCCACTTGATTTTGGAGGGA. The CFX Connect Real-Time PCR Detection System (Bio-Rad) was used to perform the PCR, which consisted of one cycle at 95 °C for 30 s, followed by 39 cycles of 95 °C for 5 s and 60 °C for 45 s. A melting analysis was performed after PCR to assess amplification specificity. The ∆/∆-Ct method was used to quantify relative mRNA expression, with *GAPDH* mRNA serving as a loading control.

### Cohort and MRI analysis

All study protocols were approved by the institutional review board of Tohoku University (approval number: 2023-0026) and National Center of Neurology and Psychiatry (approval number: A2023-081), and written informed consent was obtained from all participants prior to inclusion in the study, in accordance with the Declaration of Helsinki. Structural magnetic resonance imaging (MRI) data were acquired using a Philips Ingenia 3.0T scanner (Philips Healthcare). T1-weighted images were obtained using a 3D MPRAGE sequence in the sagittal plane and the following parameters: repetition time (TR) = 11 ms, echo time (TE) = 5.2 ms, inversion time (TI) = 1,068.3 ms, flip angle = 8°, field of view (FOV) = 256 mm, matrix size = 368 × 368, and slice thickness = 0.7 mm. The total scan time was 319 s. No additional MRI sequences were used. Images underwent N4 bias field correction, followed by a series of preprocessing steps including segmentation, normalization, and spatial smoothing using an 8-mm full-width at half maximum (FWHM) isotropic Gaussian kernel. Voxel-based morphometry (VBM) was performed to quantify regional gray matter volume. Image preprocessing and analysis were performed using SPM12 (Wellcome Centre, London, UK) and CAT12 toolbox (v12.8) implemented in MATLAB (MathWorks, USA). Group comparisons were performed using simple linear regression models, with age and intracranial volume included as covariates. A total of *n* = 160 participants were included (dimemorfan group: *n* = 80; control group: *n* = 80). The control participants were selected to match the drug groups in age and sex distribution. The details were listed in Fig. 5B. Subjects showing incomplete brain coverage were excluded from the analysis.

### Statistical analysis

Statistical analyses were performed with GraphPad Prism 10 (GraphPad software) or R by unpaired 2-tailed Student’s *t* tests, one-way analysis of variance (ANOVA) followed by Tukey’s post-test, 2-way ANOVA followed by Bonferroni’s post-test, and simple linear regression analysis. Data are shown as mean ± standard error of the mean (SEM). Differences under *P* < 0.05 were considered significant.

## Results

### Establishment of remyelination-on-a-microfiber platform using neurite mimic microfiber

**Fig. 1.**
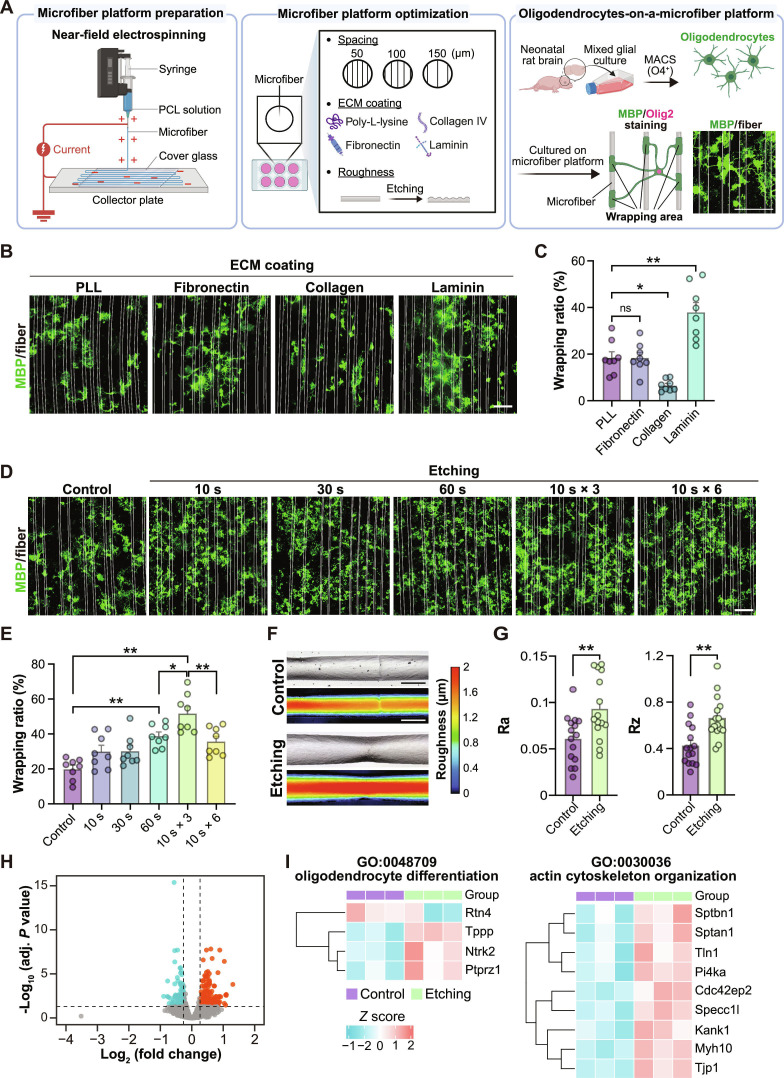
Development of a remyelination-on-a-microfiber platform. (A) Schematic illustration of a microfiber platform to evaluate remyelination. (B) Representative images of rat oligodendrocytes cultured on a microfiber platform. Cells were labeled with MBP (green). Fibers were coated with the indicated matrix. (C) Quantification of the myelin wrapping ratio in (B) (*n* = 8). (D) Representative images of rat oligodendrocytes cultured on a microfiber platform. Cells were labeled with MBP (green). Fibers were etched by RF plasma with the indicated conditions. (E) Quantification of the myelin wrapping ratio in (D) (*n* = 8). (F) Surface morphology of fibers before (control) or after applying three 10-s plasma etchings (etching). (G) Quantification of the arithmetic mean roughness (Ra) and the maximum height of the profile (Rz) in (F) (*n* = 15). (H) Volcano plot showing the differentially expressed genes (DEGs) of rat oligodendrocytes cultured on control or etching fibers. Blue and red colors indicate down-regulated and up-regulated genes, respectively. (I) Heatmap of DEGs enriched in the indicated GO terms. Scale bars, 100 μm (B and D) and 10 μm (F). Data are represented as mean ± SEM. *P* values were determined by one-way ANOVA followed by Tukey’s test (C and E) or unpaired *t* test. *P < 0.05; **P < 0.01; ns, no significant difference.

In this study, we fabricated a platform by aligning electrospun microfibers made of PCL, an Food and Drug Administration (FDA)-approved biocompatible material, on a glass-bottom dish. We cultured the rat immature oligodendrocyte (O4-positive cells) on the microfiber platform. After culturing, the cells were stained with MBP, a standard marker used to visualize the myelin sheath expressed by MOLs, and the MBP-positive area aligned along the fiber was defined as “wrapping” (Fig. 1A). We first explored the optimal interfiber spacing within a device to promote wrapping behavior, because a single oligodendrocyte is capable of forming myelin sheaths of approximately 100 to 200 μm in length [24]. Among the various configurations tested, aligned microfibers spaced at 50 μm intervals showed a higher rate of oligodendrocyte wrapping compared with other groups (Fig. S1B and C), without affecting the MBP-positive area or the number of oligodendrocyte-lineage (Olig2-positive) cells (Fig. S1D and E). We then asked whether chemical properties, such as molecular cues on the surface, regulate oligodendrocyte wrapping. Since oligodendrocyte development is known to be regulated by ECM components [25], we coated the microfiber with representative ECM proteins: collagen, fibronectin, or laminin. Among these, laminin coating markedly enhanced the wrapping rate (Fig. 1B and C), with no differences in MBP-positive cells or the number of Olig2-positive cells compared to control (PLL coating) (Fig. S2A to C). We then assessed whether physical surface properties are also involved in the efficiency of oligodendrocyte wrapping, because the surface of the neurite has topographical irregularities [26]. Among the tested conditions, wrapping efficiency was highest with the treatment involving three 10-s plasma etchings (Fig. 1D and E) without a change in MBP-positive areas and the number of Olig2-positive cells (Fig. S3A to C). We confirmed the microfiber morphology by SEM (Fig. S4A) and elevation of surface roughness parameters (Ra and Rz) in this etching condition (Fig. 1F and G), indicating enhanced surface irregularity of microfibers.

We next examined whether this culture condition enhanced oligodendrocyte wrapping at the molecular level. Transcriptomic analysis of oligodendrocytes cultured on the microfiber platform detected 87 down-regulated and 257 up-regulated genes compared with the control (Fig. 1H and Fig. S5A and B). GO enrichment analysis of these DEGs showed altered expression, in the microfiber platform-cultured cells, of genes required for promoting myelin repair (“oligodendrocyte differentiation”, GO:0048709) and for cell shape remodeling (“actin cytoskeleton organization”, GO:0030036) (Fig. 1I). More specifically, analysis based on known oligodendrocyte marker genes in the mouse CNS [27] revealed that cells cultured on etched fibers exhibited increased expression of genes associated with myelin-forming oligodendrocytes (MFOLs), such as *Clic4* and *Bmp1*, as well as genes related to MOLs, including *Ppp1r16b*, *Hapln2*, *Opalin*, *Kana1*, *Prickle1*, *Daam1*, and *Galnt6* (Fig. S5C). In addition, GO analysis and IPA of the DEGs revealed enrichment of terms related to cytoskeletal regulation, including activation of small guanosine triphosphatases (GTPases) (Fig. S5D to F). These data suggest that oligodendrocytes cultured on the microfiber platform exhibit molecular features characteristic of wrapping.

### Microfiber platform-based drug screening for wrapping-promoting compounds

**Fig. 2.**
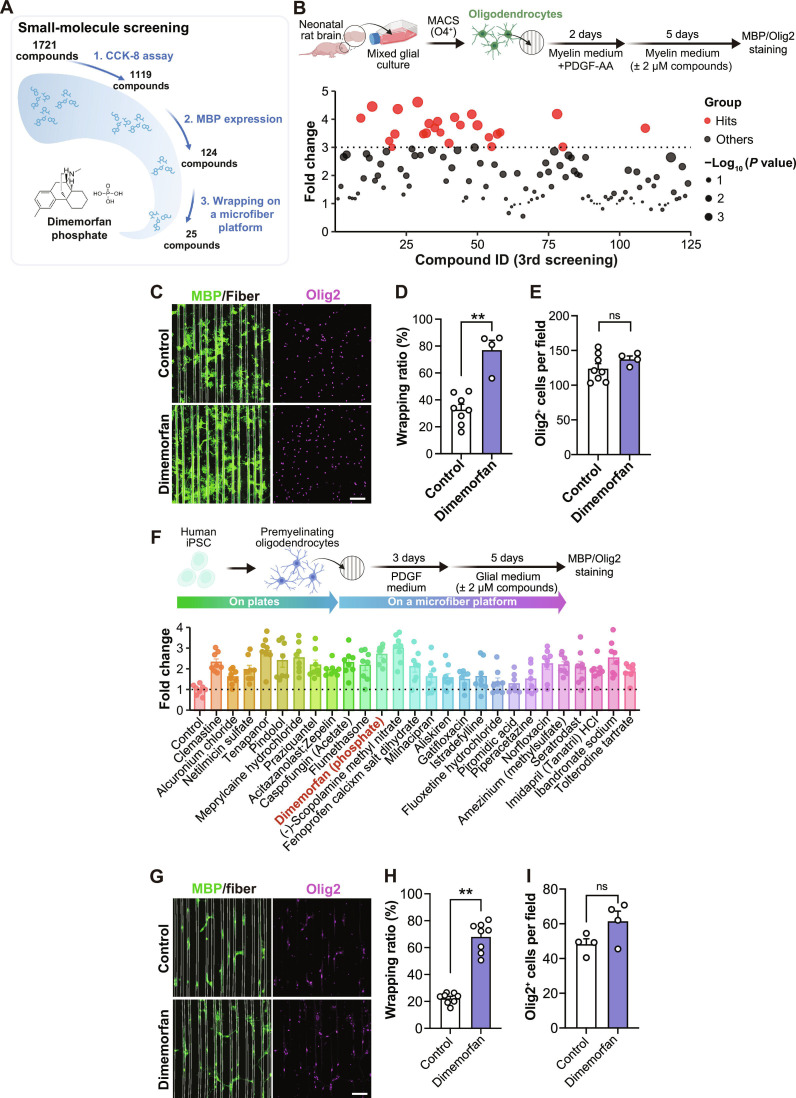
Drug screening on a microfiber platform. (A) Schematic illustration of the drug screening processes. The number of compounds was sorted by the criteria described in Materials and Methods. (B) Fold change of the wrapping ratio of the compound-treated rat oligodendrocytes relative to the control. Cells were treated with 2 μM of each compound for 5 d. (C) Representative image of rat oligodendrocytes cultured on a microfiber platform. Cells were labeled with MBP (green) and Olig2 (magenta). (D) Quantification of the myelin wrapping ratio (*n* = 8 for control, 4 for dimemorfan). (E) Quantification of Olig2^+^ cell numbers (*n* = 8 for control, 4 for dimemorfan). (F) Fold change of the wrapping ratio of the compound-treated hiPSC-derived oligodendrocytes relative to the control. Cells were treated with 2 μM of each compound for 5 d. (G) Representative image of hiPSC-derived oligodendrocytes cultured on a microfiber platform. Cells were labeled with MBP (green) and Olig2 (magenta). (H) Quantification of the myelin wrapping ratio (*n* = 8 for each). (I) Quantification of Olig2^+^ cell number (*n* = 4 for each). Scale bars, 100 μm. Data are represented as mean ± SEM. *P* values were determined by one-way ANOVA followed by Tukey’s test (B and F) or an unpaired *t* test (D, E, H, and I). **P < 0.01; ns, no significant difference.

We next investigated whether the microfiber platform could be used to screen for compounds that promote oligodendrocyte wrapping (Fig. 2A). We used U.S. FDA-approved 1,721 compounds with well-characterized mechanisms of action. The advantages of using such a library are twofold: first, the known mechanisms of action may provide insights into the molecular basis of wrapping; second, because these compounds have been previously administered to humans, existing clinical data may help infer their effects on the human brain. Before screening for wrapping-promoting effects, we assessed the impact of each compound on cell proliferation using the human oligodendrocyte cell line MO3.13. From this initial screening, 1,119 compounds that did not affect cell proliferation (fold change between 0.9 and 1.1 compared to the control) were selected by CCK-8 assay (Fig. S6A). Next, we tested whether these selected compounds promote oligodendrocyte differentiation, a prerequisite for wrapping. We immunocytochemically assessed MBP expression for each compound in culture and then proceeded to wrapping evaluation with the top 124 compounds exhibiting >2-fold higher MBP levels than the control (Fig. S6B). Of the tested compounds using rat primary O4-positive cells, 25 compounds exhibited a wrapping ratio more than twice that of the control (Fig. 2B to E), with no significant differences in the number of oligodendrocytes (Fig. S6C). For the top 25 compounds, we also performed a wrapping assay using human iPSC-derived oligodendrocytes (Fig. 2F). Among these, we focused on dimemorfan (Fig. 2G to I), given prior reports of its neuroprotective effects in a rat model of cerebral ischemia [28]. Dimemorfan is known to exert its pharmacological effects through activation of the sigma-1 receptor [15]. Therefore, we investigated whether dimemorfan promotes oligodendrocyte wrapping through the sigma-1 receptor. Silencing *SIGMAR1* in hiPSC-derived oligodendrocytes prevented dimemorfan-mediated oligodendrocyte wrapping (Fig. S7A to C). There is no significant difference in basal wrapping rate and Olig2-positive cell number by the suppression of *SIGMAR1* alone (Fig. S7B and D). These findings indicate that the pro-wrapping effect of dimemorfan is mediated, at least in part, through activation of the sigma-1 receptor.

**Fig. 3.**
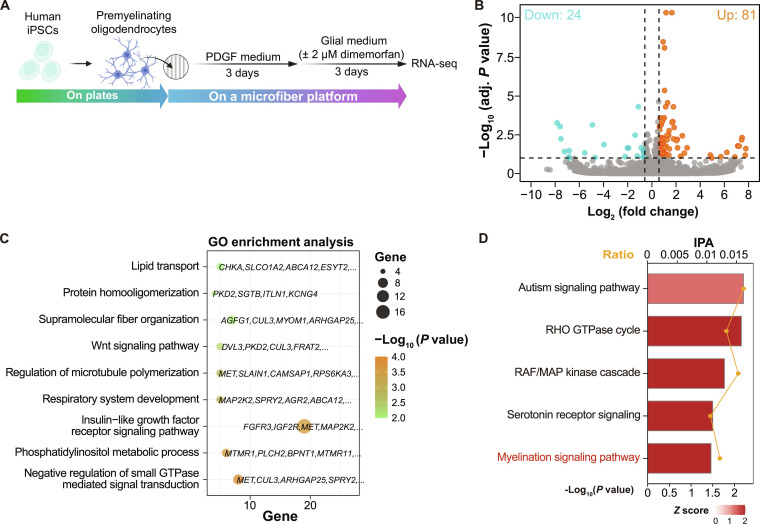
Transcriptomic profiles of wrapping-promoting compound-treated hiPSC-derived oligodendrocytes. (A) Schematic illustration of hiPSC-derived oligodendrocytes used for RNA-seq analysis. (B) Volcano plot showing the DEGs between the groups. hiPSC-derived oligodendrocytes were treated with dimemorfan for 3 d. Orange and blue spots represent up-regulated or down-regulated genes, respectively. (C) GO enrichment analysis of DEGs. (D) Ingenuity Pathway Analysis (IPA) showing the top 5 activation pathways.

As the compound identified through our platform-based screening promoted the wrapping of hiPSC-derived oligodendrocytes, we next sought to determine how it affects the transcriptomic profiles of human cells, since myelin wrapping involves not only morphological changes but also molecular alterations. We therefore tested the effect of dimemorfan in hiPSC-derived oligodendrocyte (Fig. 3A). Transcriptional analysis revealed 81 up-regulated and 24 down-regulated DEGs in the hiPSC-derived oligodendrocyte treated with dimemorfan (Fig. 3B). GO analysis of these DEGs revealed enrichment of terms associated with myelination, such as “lipid transport” and “regulation of microtubule polymerization” (Fig. 3C). Pathway analysis using IPA identified notable activation of the myelination signaling pathway (Fig. 3D). Among the DEGs, genes known to be involved in myelin formation, such as *MAP2K2*, *FGFR3*, *PRKAR2B*, and *IGF2R*, were also up-regulated (Fig. S8A). GSEA of the whole gene set using Hallmark gene sets indicated enrichment of pathways such as fatty acid metabolism and oxidative phosphorylation, which were associated with lipid biosynthesis and adenosine triphosphate (ATP) production required for myelin formation and maintenance (Fig. S8B to D). These findings support our microfiber platform-identified wrapping-promoting compounds acting on remyelination in human cells at the molecular level.

### Evaluation of wrapping-promoting compound in demyelination mouse models

**Fig. 4.**
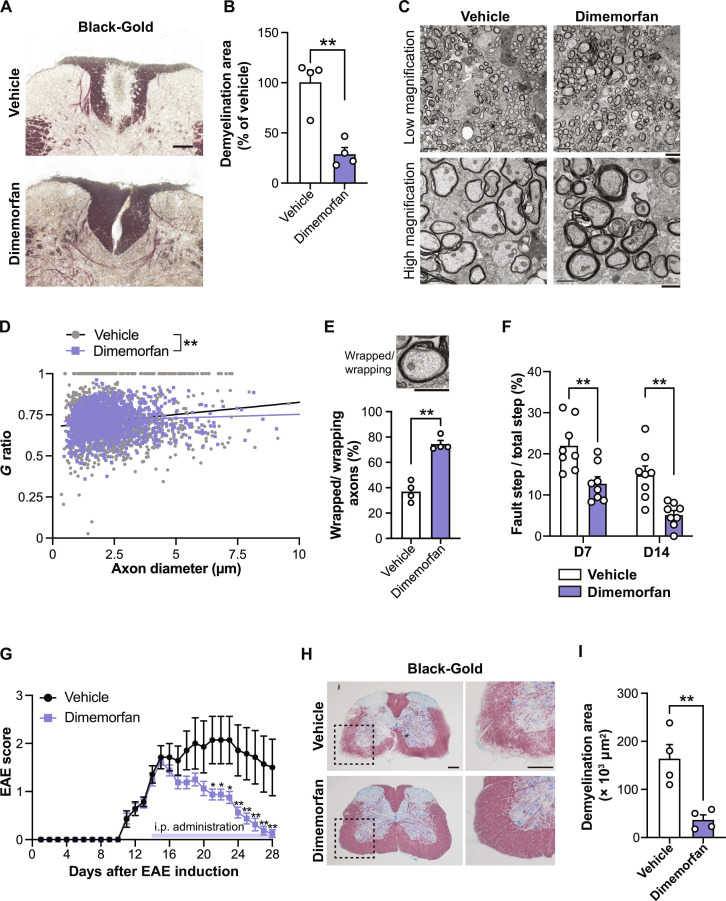
Validation of the effects of wrapping-promoting compound in the demyelination mouse model. (A) Representative images of spinal cord sections stained with Black-Gold. Mice received daily intraperitoneal injections of vehicle or dimemorfan from 3 to 14 d after LPC injection. (B) Quantification of Black-Gold-negative (demyelinating) area in the dorsal spinal cord (*n* = 4). (C) Representative electron microscope image of spinal cord sections. (D) Scatter plot of *g* ratio of individual axons in the spinal cord of mice treated with vehicle (gray) and dimemorfan (purple). At least 600 axons were quantified per mouse (*n* = 4). (E) Quantification of wrapping axons in the dorsal spinal cord (*n* = 4). (F) Quantification of the motor function evaluated by the ladder walk test. Evaluations were performed on day 7 (D7) and day 14 (D14) after LPC injection (*n* = 8). (G) Quantitation of EAE score. Mice received daily intraperitoneal injections of vehicle or dimemorfan from 14 to 28 d after EAE induction (*n* = 7 for vehicle, 8 for dimemorfan). (H) Representative images of spinal cord sections from EAE mice. Sections were stained with Black-Gold. Toluidine Blue O (blue) indicates neuronal cell bodies. Right panels, magnified view of the dashed-line area in the left panels. (I) Quantification of Black-Gold-negative (demyelinating) area in the spinal cord of EAE mice (*n* = 4). Scale bars, 100 μm (A), 5 μm [low magnification in (C)], 1 μm [high magnification in (C) and (E)], and 200 μm (H). Data were represented as mean ± SEM. *P* values were determined by unpaired *t* test (B, E, F, and I), simple linear regression of slopes (D), and 2-way ANOVA followed by Bonferroni’s test (G). *P < 0.05, **P < 0.01.

We next investigated whether the compounds identified through our microfiber platform-based screening also exhibit therapeutic effects in vivo. To ask this, we employed a toxin (LPC)-induced demyelination model, which creates focal demyelination around the LPC injection site [20] (Fig. 4A). We treatedLPC-injected mice with dimemorfan and evaluated the demyelinating area detected by Black-Gold staining, which visualizes myelin. The mice treated with dimemorfan showed a smaller Black-Gold-negative area around the injection site of the mouse spinal cord compared to the controls (Fig. 4A and B), suggesting that dimemorfan enhanced remyelination. We then assess the effect of dimemorfan on the formation of myelin sheaths by electron microscopy (EM) analysis. The extent of myelin wrapping was quantified by the *g* ratio, defined as the inner axonal diameter divided by the total outer (axon + myelin) diameter; higher *g* ratios reflect thinner myelin. The mice treated with dimemorfan showed significantly lower values of *g* ratio compared to controls (Fig. 4C and D). We also detected that the proportion of myelinated axons was higher in the dimemorfan group than in controls (Fig. 4E). These findings suggest that our microfiber-based screening platform can successfully identify compounds that promote myelin wrapping in vivo.

We thus investigated whether treatment with the compound that enhances myelin wrapping would likewise modulate behavioral performance. LPC injection into the dorsal lower thoracic spinal cord induces motor deficits in the hindlimbs of mice, which can be evaluated using the ladder walk test [20]. We then asked whether dimemorfan treatment exerts functional benefits in LPC-injected mice. Quantitative analysis revealed that the mice with dimemorfan treatment exhibited fewer missteps compared to controls (Fig. 4F), indicating that wrapping-promoting compounds lead to improved recovery of motor function.

We also examined the effect of the wrapping-promoting compound in other disease models. EAE is a commonly used experimental model for the inflammatory demyelinating disease, MS. We monitored EAE severity using the clinical EAE score and initiated continuous administration of dimemorfan at the time when symptoms worsened. Quantitative analysis revealed that the dimemorfan-treated group showed a reduced EAE score (Fig. 4G). We also analyzed myelin formation in spinal cord tissue after behavioral observation. Histological analysis revealed that the mice treated with dimemorfan showed a smaller Black-Gold-negative area compared with that in controls (Fig. 4H and I). These findings suggest that our microfiber-based platform can identify compounds that promote tissue repair and improve behavioral outcomes across demyelinating disease models.

### Predicted effects of wrapping-promoting compound on human myelin

**Fig. 5.**
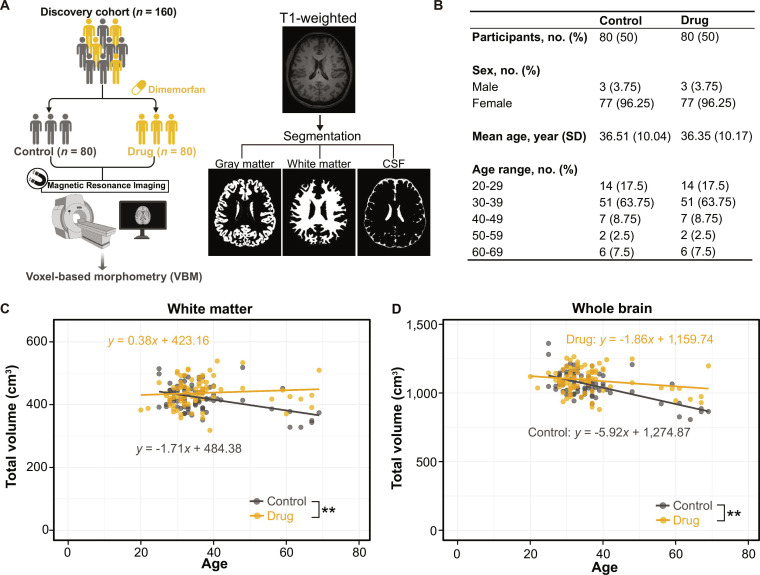
Validation of the effects of wrapping-promoting compound in human brain. (A) Workflow of voxel-based morphometry (VBM) analysis using MRI images from participants. (B) Characteristics of the participants enrolled in the MRI analyses. Participant characteristics stratified by age group and sex, as included in the MRI dataset. (C and D) Quantification of white matter volume (C) and total brain volume (D) in dimemorfan-treated (drug) and untreated (control) groups (*n* = 80). *P* values were determined by a simple linear regression model. **P < 0.01.

We finally asked about the potential effects of the wrapping-promoting compound on the human brain. We analyzed the structural MRI data from individuals with a history of dimemorfan use, drawn from the Tohoku Medical Megabank cohort (Fig. 5A). We conducted voxel-based morphometry (VBM) analysis to quantify brain tissue volume (Fig. 5B) by comparing the dimemorfan-treated group with age- and sex-matched controls. Myelin content is known to decline with aging [29], and our analysis also showed the trend of the age-related white matter volume reduction in the control group (Fig. 5C). In contrast, individuals with a history of dimemorfan use exhibited attenuated age-related white matter loss (Fig. 5C), speculating that the compound identified through our microfiber-based screening prevents the reduction of myelin volume. Individuals treated with dimemorfan dosing also showed increased total brain volumes (Fig. 5D) and greater gray matter (Fig. S9A), without changing the cerebrospinal fluid volume (Fig. S9B), compared to controls. These results suggest that our microfiber-based screening platform has the potential to identify compounds effective in promoting remyelination in the human brain.

## Discussion

Neural function is traditionally attributed to changes in neural circuits; however, non-neuronal cells, including oligodendrocytes, also play a critical role in regulating CNS activity. Myelination is considered a fundamental process that enables neural adaptation, supporting the brain’s ability to modify (learning), maintain (homeostasis), and restore (recovery from injury) its function in response to environmental changes [30]. In this study, we present a novel platform for evaluating myelination using microfibers that mimic neuronal processes. Although we primarily employed this platform for screening compounds with the potential to promote remyelination, it is also applicable to studying mechanisms of developmental myelination and myelin maintenance through turnover in the mature brain. Therefore, this platform has the potential to contribute to a broader understanding of how myelin supports normal brain function.

Our platform offers the advantage of enabling the quantification of both the extent and length of oligodendrocyte wrapping from a single 2-dimensional image. Additionally, the use of a glass-bottom substrate allows for compatibility with fluorescent reporter-based imaging and time-lapse analysis. The device is designed so that cells are seeded between microfibers, providing a uniform mechanical substrate. Given that oligodendrocytes respond sensitively to the physical properties of their environment, such a uniform seeding design ensures consistency in cellular responses, facilitating more reliable analyses. Interestingly, oligodendrocytes exhibited spontaneous wrapping even on untreated microfibers. Elucidating the molecular basis of this phenomenon could provide important insights into the intrinsic mechanisms initiating wrapping behavior in oligodendrocytes. Moreover, enhanced wrapping was observed under chemically or physically modified surface conditions. The ability to detect differences in wrapping efficiency based on surface modifications suggests that this platform may also be useful in dissecting the molecular and physical cues that modulate oligodendrocyte wrapping behavior. We further applied this platform under wrapping-favorable conditions for compound screening. Compounds that enhance wrapping under such conditions are likely to act synergistically with the cell’s intrinsic wrapping mechanisms and may therefore be promising candidates for augmenting oligodendrocyte function in chronic disease states where remyelination capacity is diminished.

From this screen, we identified dimemorfan as a compound that markedly promotes oligodendrocyte wrapping. Dimemorfan is a known antitussive agent that acts on the CNS and has an established safety profile [31], raising the possibility of drug repurposing of dimemorfan for demyelinating diseases such as MS. Furthermore, VBM analysis from human specimens indicated that individuals with a history of dimemorfan administration showed a less pronounced age-related decline in white matter volume compared with the controls, suggesting a potential protective effect against neurodegenerative disorders in which aging is a major risk factor. We found that the pro-wrapping effect of dimemorfan is mediated through the sigma-1 receptor, a known target molecule of this compound [15]. Sigma-1 receptor activation is associated with neuroprotective and neuromodulatory effects [32], suggesting that the effect of dimemorfan that improves neurological functions is not only through enhanced myelination but also via additional CNS-supportive pathways in vivo. Of note, knockdown of sigma-1 receptor expression alone (without dimemorfan treatment) did not abolish the wrapping-promoting effect, indicating that the mechanism of sigma-1 receptor-promoted wrapping is a modulatory action to enhance preexisting cell-intrinsic wrapping capabilities, which is supported by the previous findings showing that sigma-1 receptor knockout mice do not exhibit marked phenotypic differences in the brain compared to wild-type animals [33].

Looking forward, this platform has broad potential for future applications. Oligodendrocyte-mediated myelination is regulated not only through interactions with neurons but also through crosstalk with other cell types, such as microglia [34,35] and vascular endothelial cells [36]. Therefore, by establishing coculture systems with heterologous cell types, this platform may facilitate the elucidation of extracellular mechanisms governing myelin formation and maintenance. The concept that circulating cells and factors originating outside the CNS can influence neurons within the brain is well established [37,38], and similarly, remyelination by oligodendrocytes is thought to be regulated by circulating humoral factors [19,20]. Therefore, this approach could also be applied to analyze the effects of brain-external factors, such as characteristics of aging or peripheral diseases, on oligodendrocytes. Oligodendrocyte function can also be altered by disease-associated genetic changes. As demonstrated in this study using cells with targeted gene knockdown, the platform is compatible with analyses of genetically modified cells. This opens avenues for evaluating patient-derived cells or cells engineered to express disease-relevant mutations, thereby enabling disease-specific investigations into the mechanisms of myelination.

Although this study utilized a 2-dimensional culture system, oligodendrocytes, like other cell types, are known to respond differently in 3-dimensional environments. Refining the dimensionality of the culture and optimizing ECM composition may enable the reconstruction of more physiologically relevant human brain-like conditions. Such advances would improve the translational relevance of both mechanistic and drug screening studies conducted with this system. It is important to note that the wrapping phenomenon observed in our study represents an early step in the myelination process, analogous to the initial stages of membrane attachment seen in pillar-based assays. However, the length, thickness, and internodal spacing of myelin sheaths are known to critically influence conduction properties of axons. Currently, it remains unclear whether new myelin sheaths are continuously generated in vivo or whether preexisting sheaths mature over time. Future technological advancements in live imaging capable of capturing dynamic changes in myelin structure may synergize with platforms like ours to elucidate functional differences among distinct myelination modes.

While this study focused on CNS myelination, Schwann cells are responsible for myelination in the peripheral nervous system (PNS) [39]. Additionally, although the target structures differ in size, blood vessels are also wrapped by smooth muscle cells [40]. Therefore, the ability to assess cellular wrapping behaviors suggests that this platform may be applicable beyond CNS studies, potentially extending to investigations of PNS or vascular cell responses. Finally, microstructured materials such as those used in this study are not limited to use as analytical tools; they are also being explored as scaffolds for regenerative medicine [41]. In particular, fiber scaffolds have already been utilized to support axonal regeneration in the PNS [42]. Although biodegradability was not evaluated in the present in vitro study, future in vivo applications will require careful assessment of degradation kinetics and biocompatibility, as residual scaffold materials may influence local mechanical properties and cellular responses. Combining such structural platforms with gene therapy or stem cell technologies may lead to novel strategies for tissue repair and regenerative medicine in the future.

## Data Availability

All data are available in the main text or the Supplementary Materials.
