# Supplementary material for: Biomimetic Microfibers for Myelin-Enhancer Screening and Neural Regeneration
Source: Cyborg Bionic Syst. 2026 May 7;7:0565. doi: 10.34133/cbsystems.0565 (PMC13150081; doi:10.34133/cbsystems.0565)
Supplement: Supplementary 1 — Figures S1 to S9 Table S1 [file cbsystems.0565.f1.zip › Supplementary info_260219.docx]

**Table S1. Compositions of reagents and products for use in cell culture**

| **Name** | **Maker** | **Cat no** | **Final conc.** |
| --- | --- | --- | --- |
| **Myelin medium** |  |  |  |
| DMEM | Gibco | 12800-017 |  |
| B27 Supplement (50 ×) | Gibco | 12587010 | 1 × |
| N2 Supplement (100 ×) | Gibco | 17502048 | 1 × |
| N-acetylcysteine | Sigma-Aldrich | A8199 | 5 μg/mL |
| Forskolin | Sigma-Aldrich | 344270 | 5 μM |
| Penicillin-Streptomycin | Fuji film | 161-23181 | 0.5 × |
| **Myelin medium + PDGF-AA** |  |  |  |
| Myelin medium |  |  |  |
| PDGF-AA | PeproTech | 100-13A | 10 ng/mL |
| **Basel medium**  DMEM/F12 | Sigma-Aldrich | D0547 |  |
| GlutaMAX™ Supplement | Gibco | 35050061 | 1 × |
| MEM Non-Essential Amino Acids | Gibco | 11140050 | 1 × |
| 2-Mercaptoethanol | Gibco | 21985023 | 1 × |
| Penicillin-Streptomycin | Fuji film | 161-23181 | 1 × |
| **Neural induction medium**  mTeSR1-cGMP | Veritas | ST-85850 |  |
| SB431542 | Tokyo Chemical Industry | B4003 | 10 M |
| LDN193189 | Reprocell | 04-0074 | 250 nM |
| Retinoic acid | Sigma-Aldrich | R2625 | 100 nM |
| **N2 medium**  Basel medium |  |  |  |
| N2 Supplement (100 ×) | Gibco | 17502048 | 1 × |
| **N2B27 medium**  Basel medium |  |  |  |
| Insulin solution human | Sigma-Aldrich | I9278 | 25 μg/mL |
| N2 Supplement (100 ×) | Gibco | 17502048 | 1 × |
| B27 Supplement (50 ×) | Gibco | 12587010 | 1 × |
| Retinoic acid | Sigma-Aldrich | R2625 | 100 nM |
| Smoothened Agonist (SAG) | Sigma-Aldrich | 566660 | 1 μM |
| **PDGF medium**  Basel medium |  |  |  |
| N2 Supplement (100 ×) | Gibco | 17502048 | 1 × |
| B27 Supplement (50 ×) | Gibco | 12587010 | 1 × |
| Recombinant Human PDGF-AA | R&D Systems | 221-AA | 10 ng/mL |
| Recombinant Human IGF-I | R&D Systems | 291-G1 | 10 ng/mL |
| Recombinant Human HGF | R&D Systems | 294-HGN | 5 ng/mL |
| Recombinant Human NT-3 | WAKO | 146-09231 | 10 ng/mL |
| T3 | Sigma-Aldrich | T6397 | 60 ng/mL |
| Biotin | Sigma-Aldrich | B4639 | 100 ng/mL |
| cAMP | Sigma-Aldrich | D0260 | 1 μM |
| Insulin solution human | Sigma-Aldrich | I9278 | 25 μg/mL |
| **Glial medium**  Basel medium |  |  |  |
| N2 Supplement (100 ×) | Gibco | 17502048 | 1 × |
| B27 Supplement (50 ×) | Gibco | 12587010 | 1 × |
| HEPES | Sigma-Aldrich | H4034 | 10 mM |
| T3 | Sigma-Aldrich | T6397 | 60 ng/mL |
| Biotin | Sigma-Aldrich | B4639 | 100 ng/mL |
| cAMP | Sigma-Aldrich | D0260 | 1 μM |
| Insulin solution human | Sigma-Aldrich | I9278 | 25 μg/mL |
| Ascorbic acid | Tokyo Chemical Industry | A0537 | 20 μg/mL |
| **Others** |  |  |  |
| Anti-O4 MicroBeads | Miltenyi Biotec | 130-094-543 |  |
| LS Columns | Miltenyi Biotec | 130-042-401 |  |


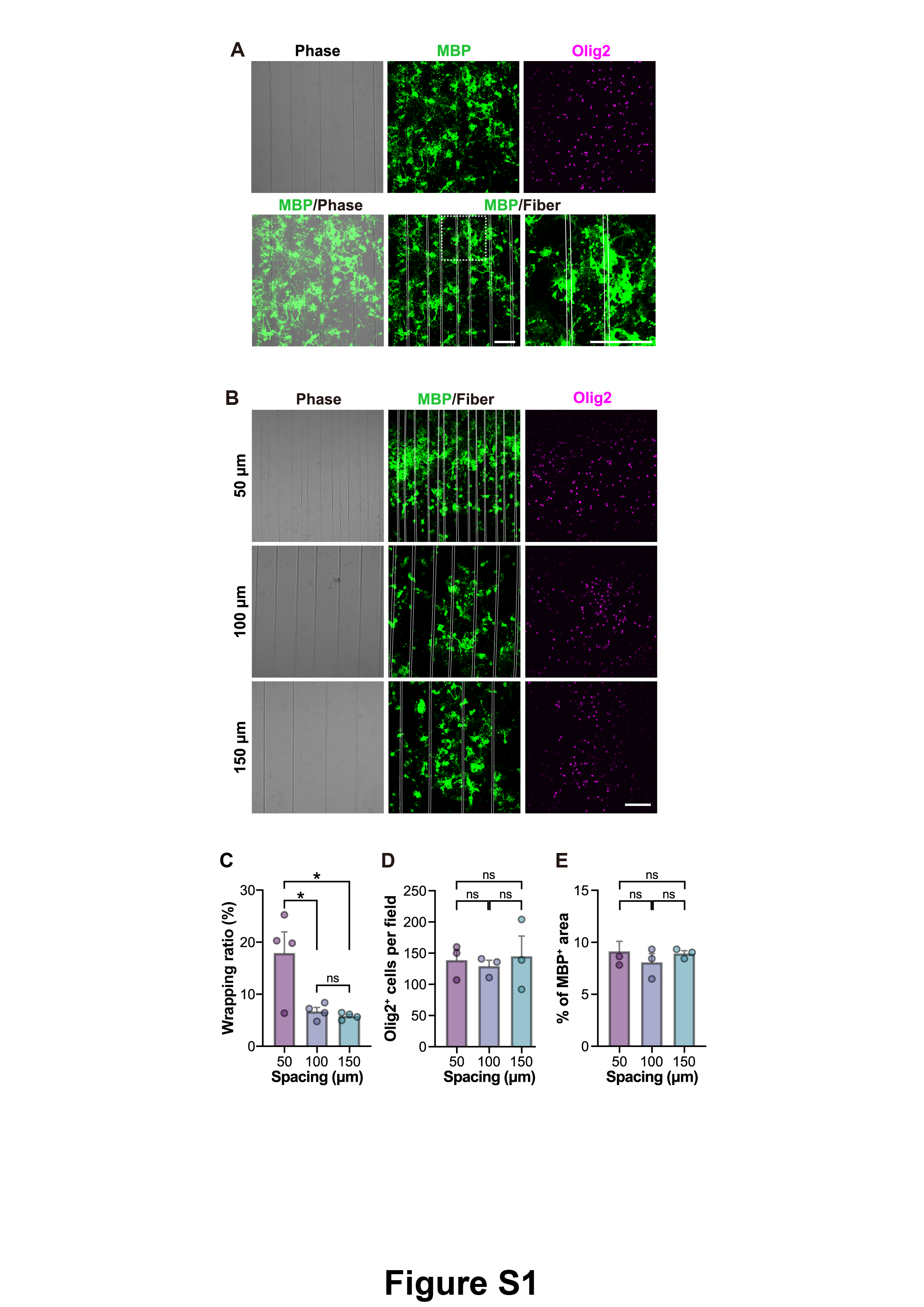


**Fig. S1: Optimization of the fiber interval of the microfiber platform**

(**A**) Representative images of rat oligodendrocytes cultured on a microfiber platform. Cells were labeled with MBP (green) and Olig2 (magenta). Fibers were shown in the phase contrast images. Fibers were lined with indicated intervals. (**B**) Representative images of rat oligodendrocytes cultured on a microfiber platform with the indicated fiber interval. Cells were labeled with MBP (green) and Olig2 (magenta). (**C**) Quantification of the myelin wrapping ratio (n = 4). (**D**) Quantification of MBP^+^ area (n = 3). (**E**) Quantification of Olig2^+^ cell numbers (n = 3). Scale bars: 100 µm. Data are represented as mean ± SEM. *P* values were determined by one-way ANOVA followed by Tukey’s test. * *p* < 0.05, ns, no significant difference.


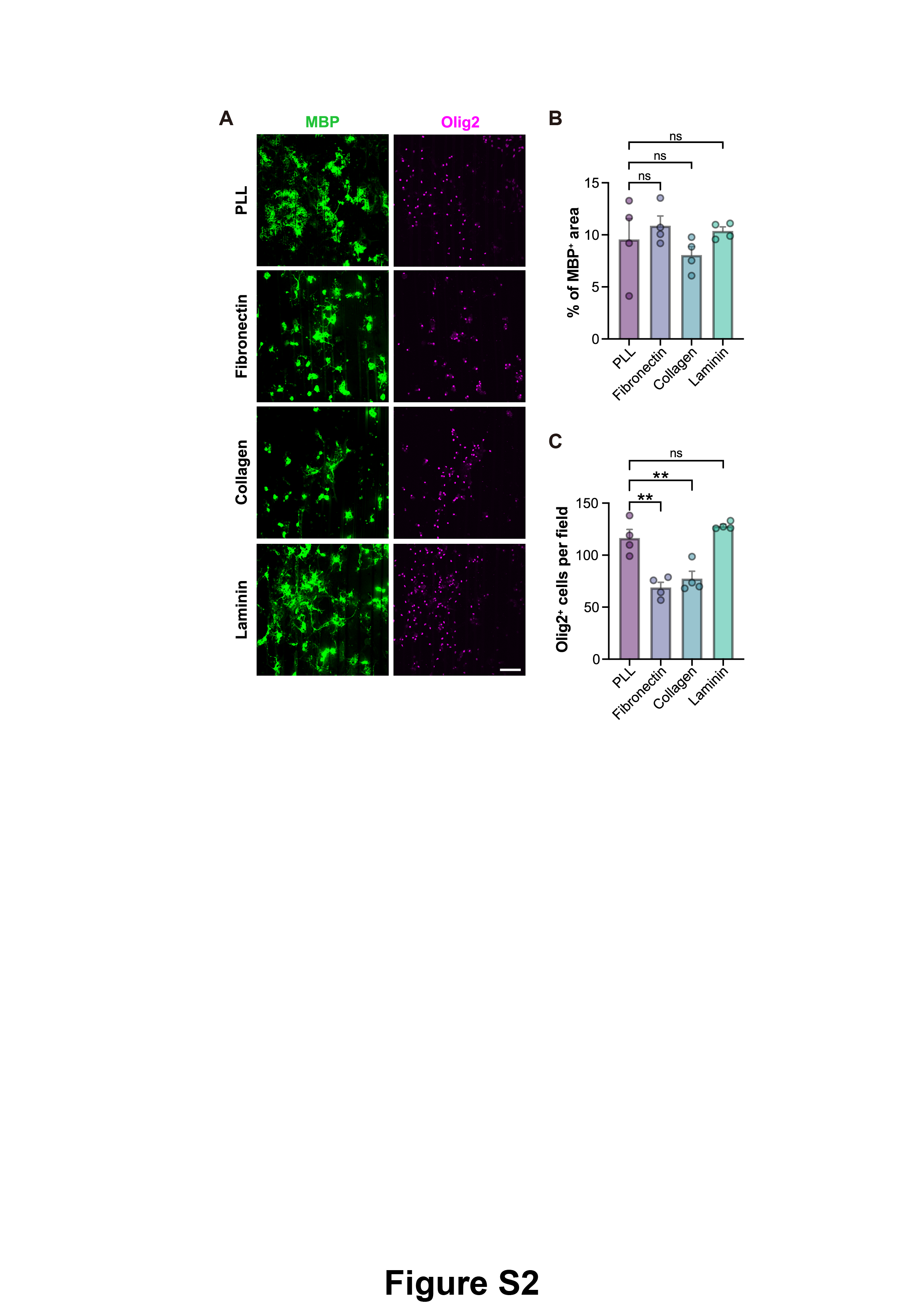


**Fig. S2: Matrix coating effects on the oligodendrocyte growth**

(**A**) Representative images of rat oligodendrocytes cultured on a microfiber platform. Cells were labeled with MBP (green) and Olig2 (magenta). Microfiber platforms were pre-coated with the indicated matrix. (**B**) Quantification of MBP^+^ area (n = 4). (**C**) Quantification of Olig2^+^ cell numbers (n = 4). Scale bar: 100 µm. Data are represented as mean ± SEM. *P* values were determined by one-way ANOVA followed by Tukey’s test. ** *p* < 0.01, ns, no significant difference.

**
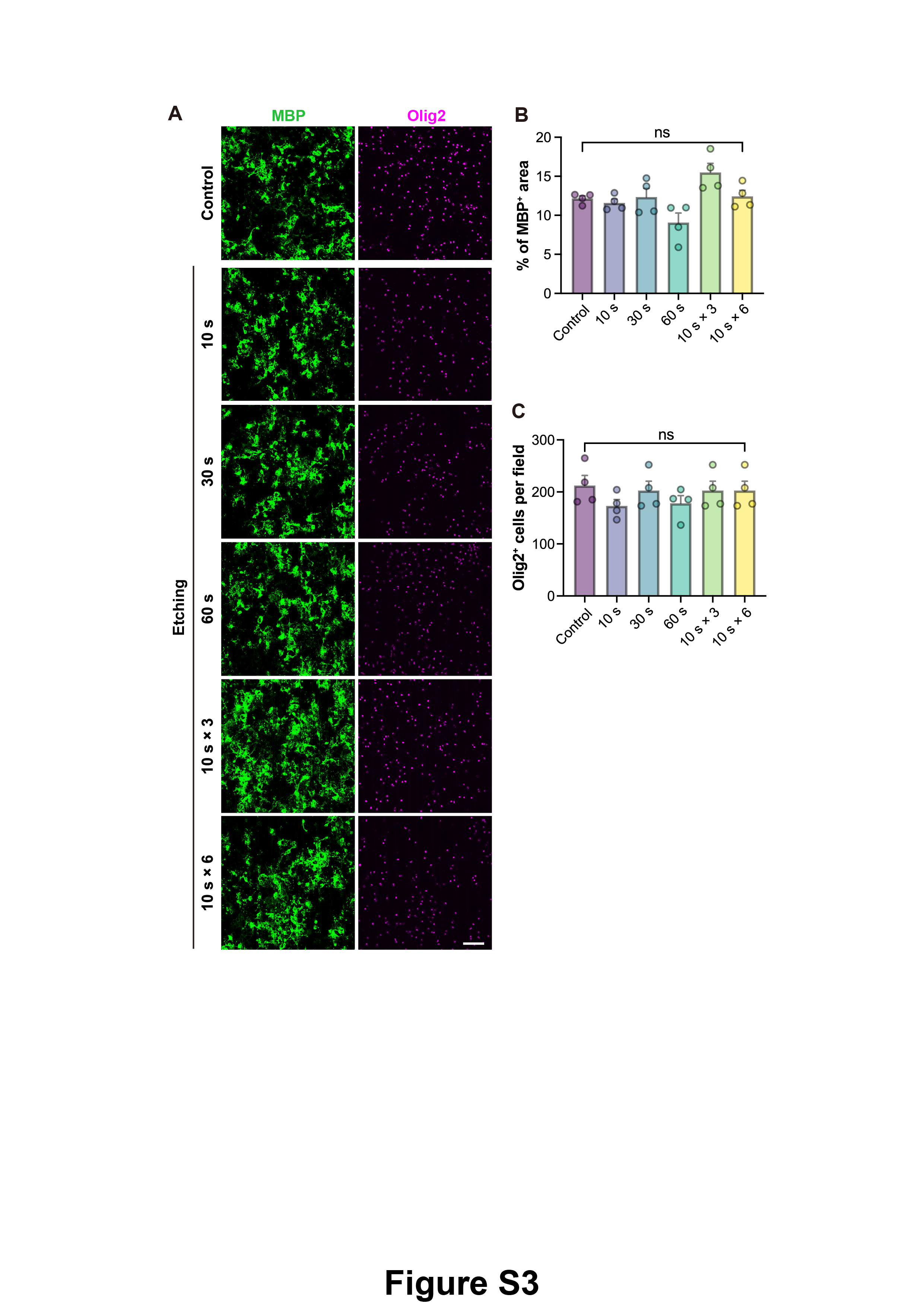
**

**Fig. S3: Fiber etching effects on the oligodendrocyte growth**

(**A**) Representative images of rat oligodendrocytes cultured on a microfiber platform. Cells were labeled with MBP (green) and Olig2 (magenta). Fibers were etched by the indicated conditions. (**B**) Quantification of MBP^+^ area (n = 4). (**C**) Quantification of Olig2^+^ cell numbers (n = 4). Scale bar, 100 μm. Data are represented as mean ± SEM. *P* values were determined by one-way ANOVA followed by Tukey’s test. ns, no significant difference.

**Fig. S4: Surface morphology of microfibers**

Representative SEM images of microfibers with (etching) or without (control) applying three 10-second plasma etchings. Scale bar, 5 µm.


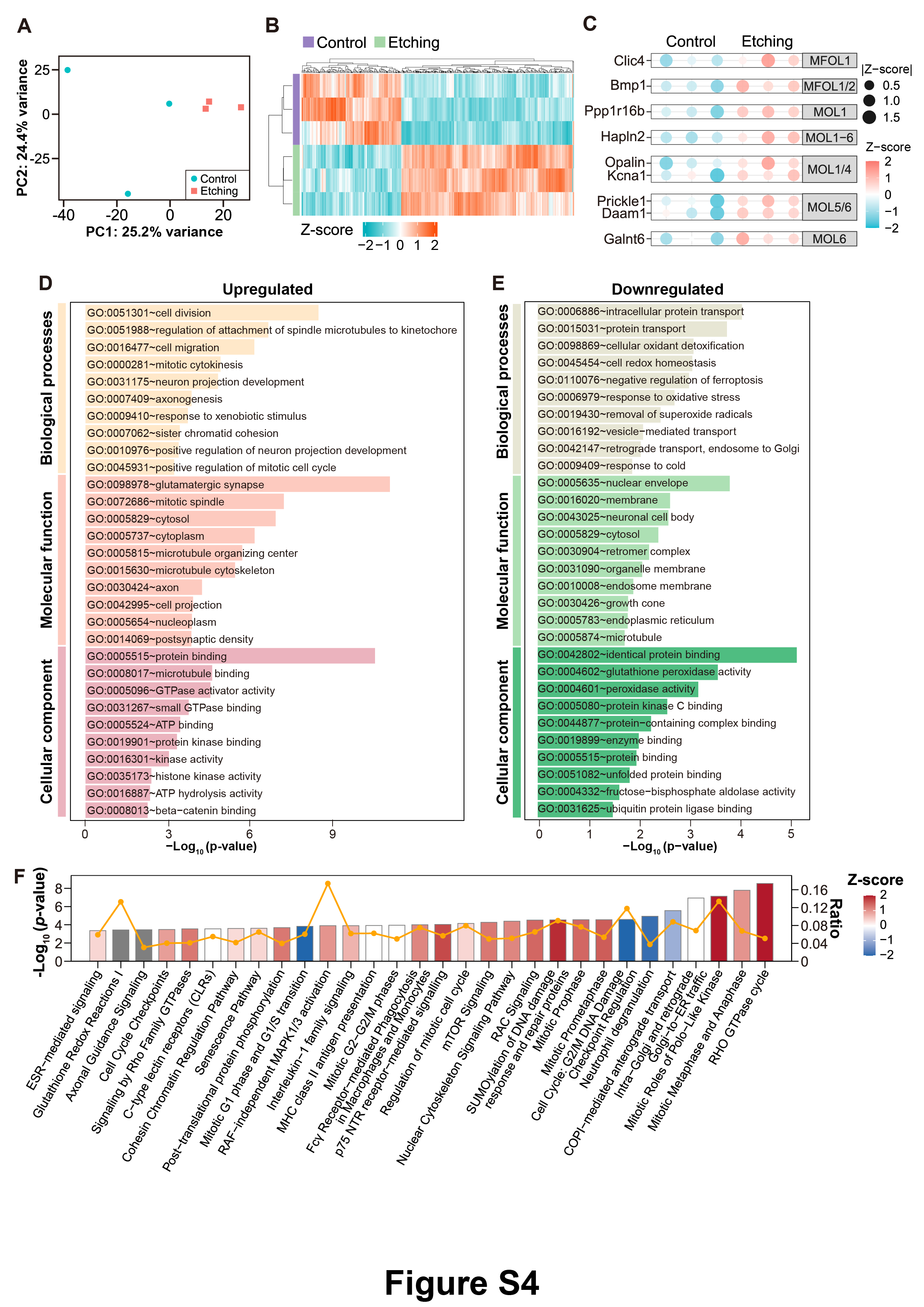


**Fig. S5: Transcriptomic profiles of oligodendrocytes cultured on the microfiber platform**

(**A**) PCA plot of transcriptomic profiles. (**B**) Heatmap of DEGs. (**C**) Upregulated genes in the oligodendrocyte cultured on etching fibers within identified myelin-forming oligodendrocytes (MFOL) and mature oligodendrocytes (MOL). (**D** and **E**) The top 10 enriched GO terms of upregulated (D) and downregulated (E) genes in oligodendrocytes cultured on etching fibers were identified in the categories of biological process, molecular function, and cellular component. (**F**) Top 30 related pathways enriched in DEGs identified by Ingenuity pathway analysis (IPA).

**
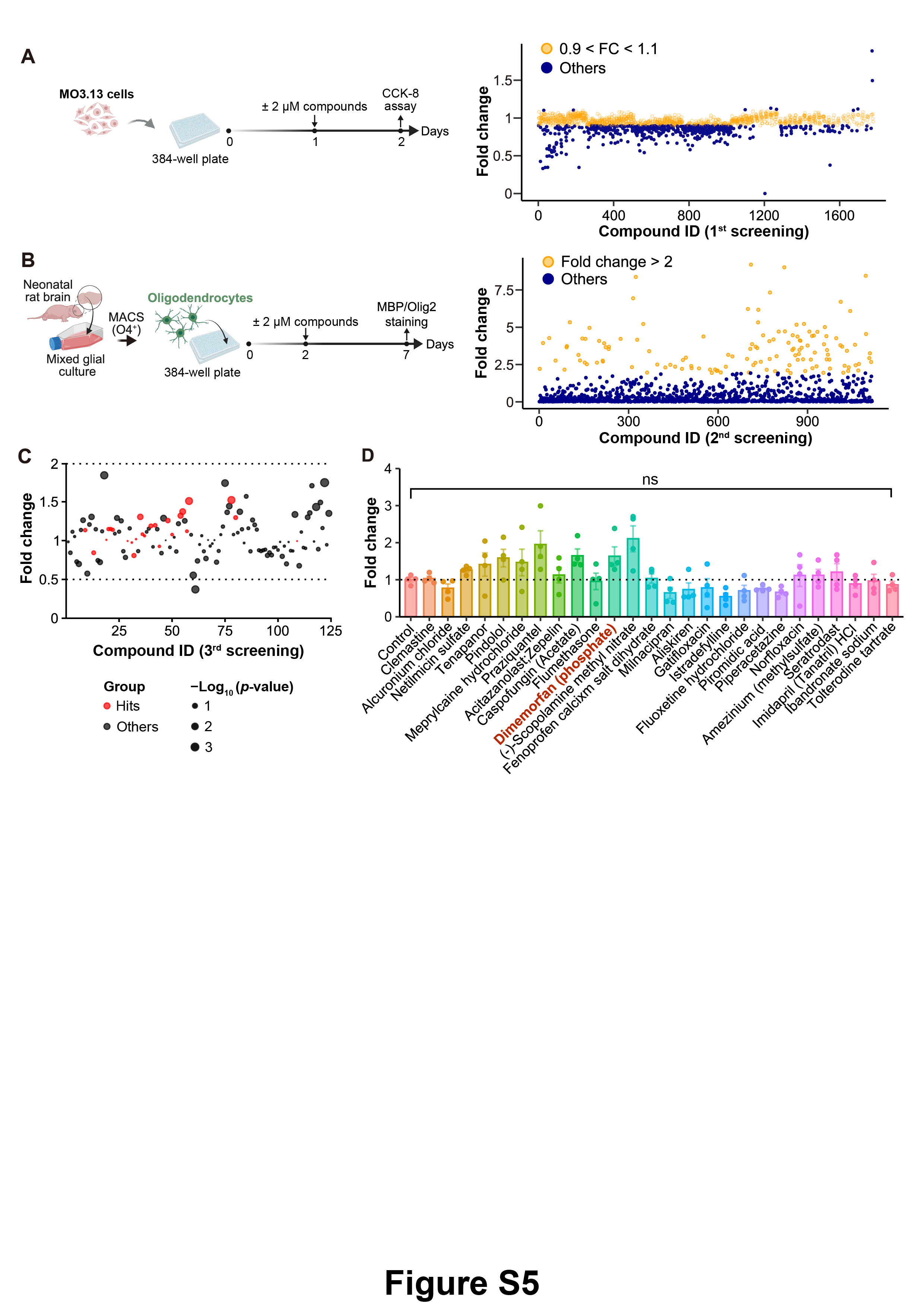
**

**Fig. S6: Prescreening stage before microfiber platform-based screening**

(**A**) Relative cell proliferation of the MO3.13 cells (human oligodendrocyte cell line) treated with 2 μM of each compound. Cell proliferation was evaluated by CCK-8 assay. Orange spots indicate fold changes within the range of 0.9 to 1.1. (**B**) Relative MBP expression in rat oligodendrocytes treated with 2 μM of each compound. Orange spots indicate fold changes greater than 2. (**C**) Quantification of Olig2^+^ cell number in the rat oligodendrocyte treated with each compound. (**D**) Quantification of Olig2^+^ cell number in hiPSC-derived oligodendrocytes treated with 2 μM of each compound. Data are represented as the mean. *P* values were determined by one-way ANOVA followed by Tukey’s test. ns, no significant difference.


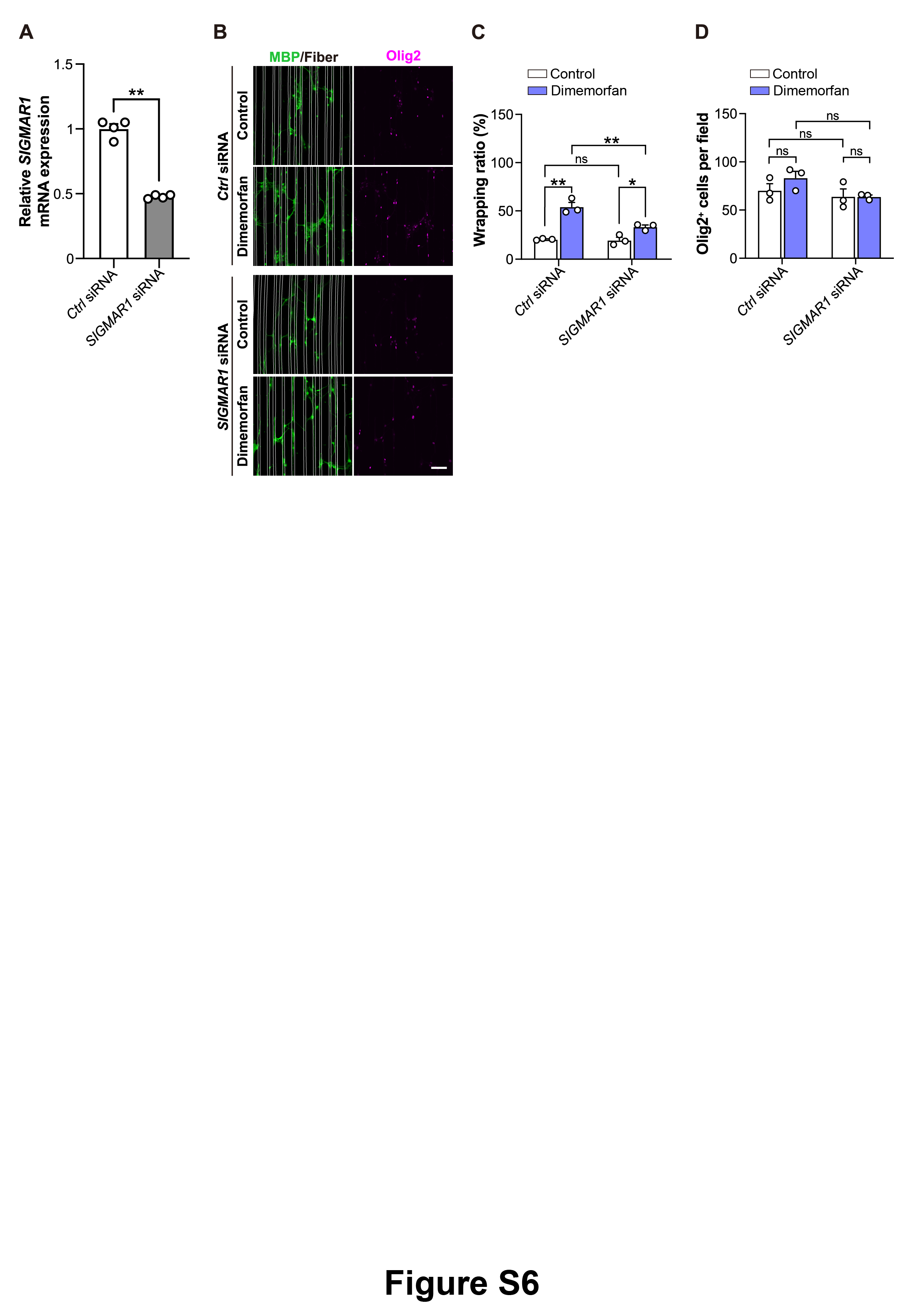


**Fig. S7: Dimemorfan promotes myelin wrapping via Sigma-1 receptor**

(**A**) Relative expression of SIGMAR1 in hiPSC-derived oligodendrocyte. Cells were transfected with siRNA for SIGMAR1 or non-target control (Ctrl) (n = 4). (**B**) Representative image of hiPSC-derived oligodendrocyte cultured on the microfiber platform. Cells were transfected with siRNA for SIGMAR1 or Ctrl, and then were treated with 2 mM of Dimemorfan. Cells were labeled with MBP (green) and Olig2 (magenta). (**C**) Quantification of the myelin wrapping ratio (n = 3). (**D**) Quantification of Olig2^+^ cell numbers (n = 3). Scale bar, 100 μm. Data were represented as mean ± SEM. *P* values were determined by unpaired t-test (A) or one-way ANOVA followed by Tukey’s test (C and D). * *p* < 0.05, ** *p* < 0.01, ns, no significant difference.

**
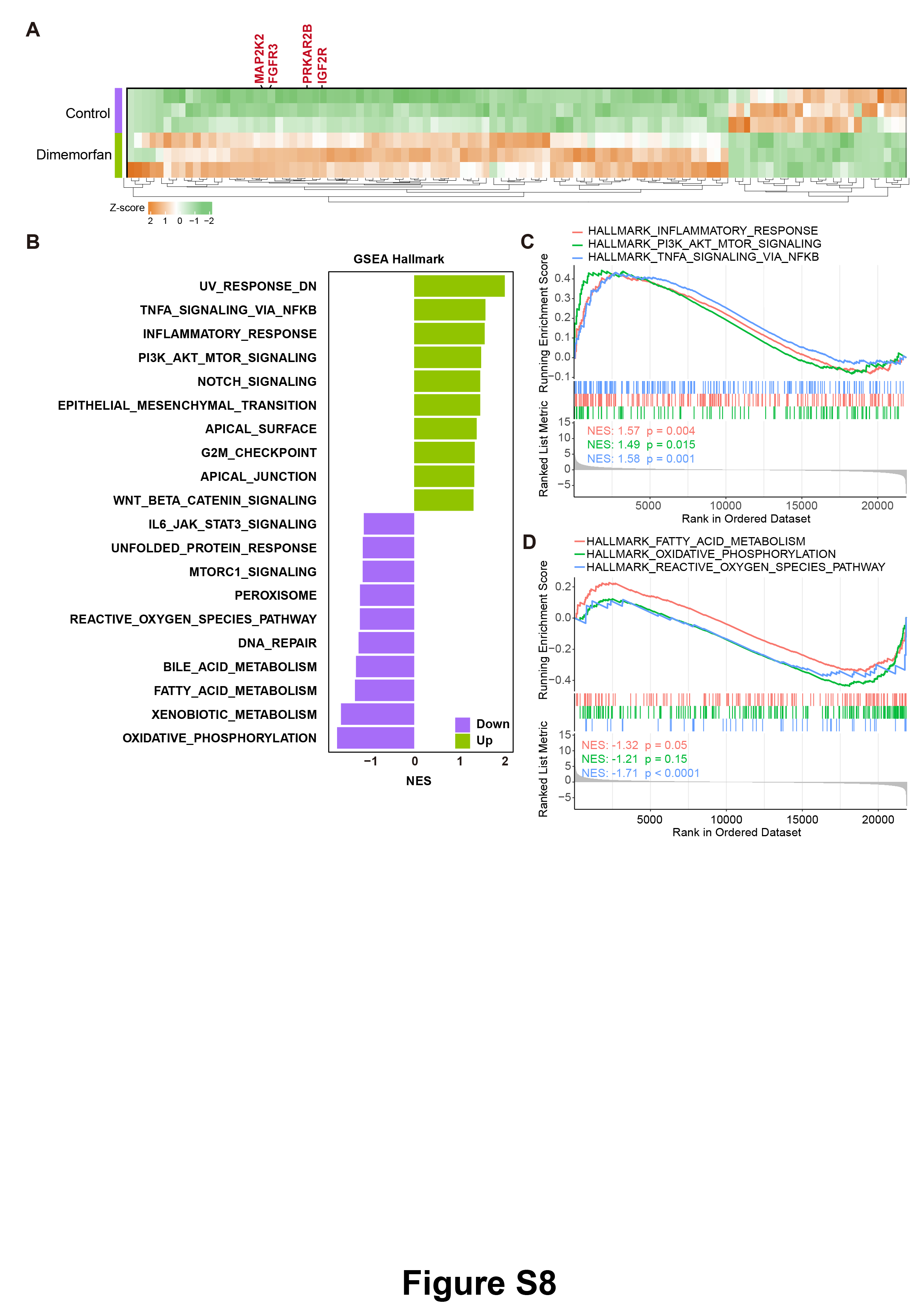
**

**Fig. S8: Transcriptomic change of hiPSC-derived oligodendrocytes treated with wrapping-promoting compound**

(**A**) Heatmap showing the DEGs in the hiPSC-derived oligodendrocyte between control and Dimemorfan treatment. (**B**) GSEA analysis showing the enrichment of hallmark pathways in the hiPSC-derived oligodendrocytes treated with Dimemorfan. (**C** and **D**) Representative GSEA plot of positive (C) and negative (D) enriched pathways.


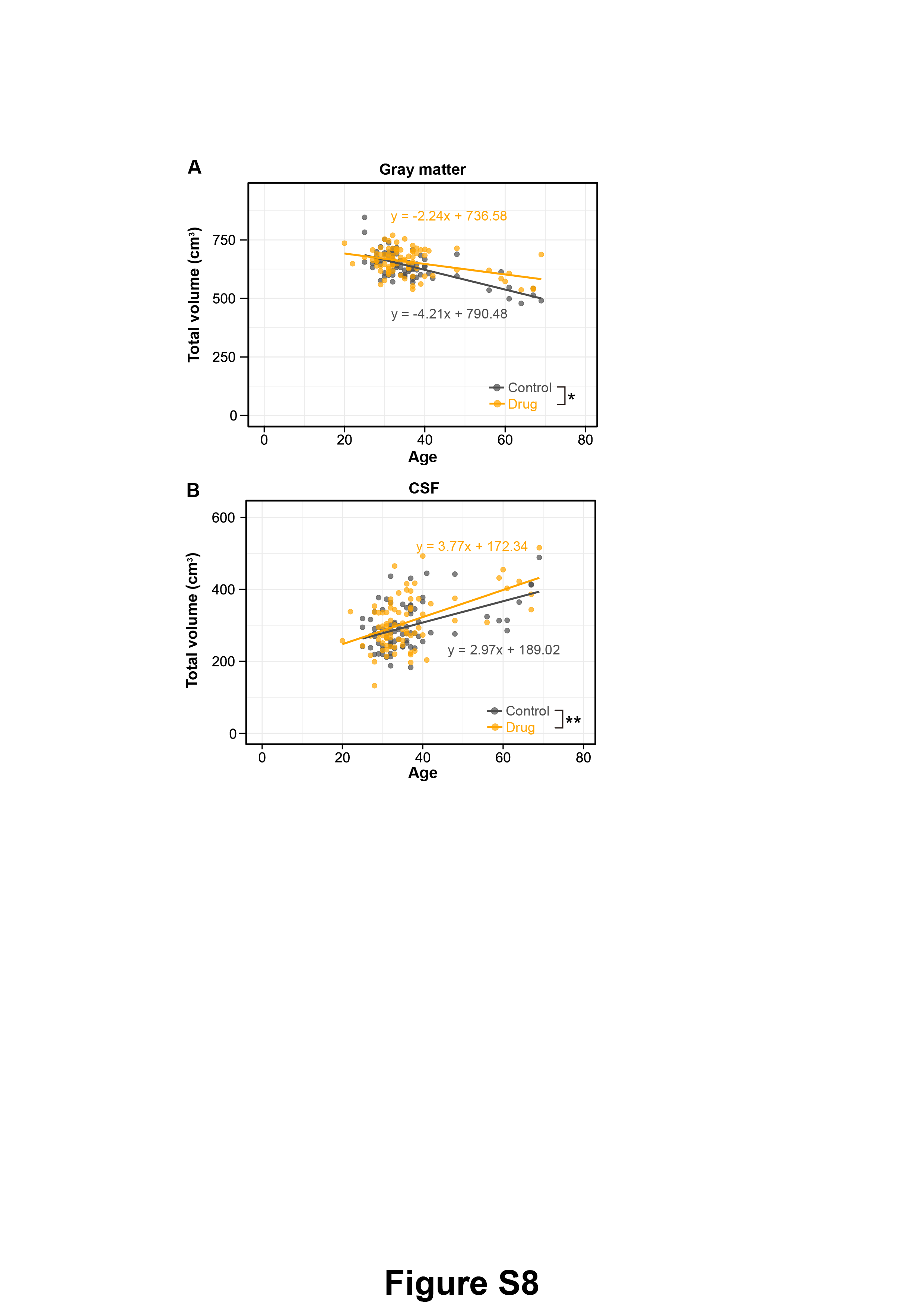


**Fig. S9: MRI image analysis in the individuals treated with Dimemorfan**

(**A** and **B**) Age-dependent change of gray matter volume (A) and CSF volume (B) among individuals with the treatment of dimemorfan (orange) or control individuals (gray) (n = 80). Data are statistically analyzed by a simple linear regression model. * *p* < 0.05, ** *p* < 0.01.
