# Supplementary figures and images for: Biomimetic Microfibers for Myelin-Enhancer Screening and Neural Regeneration
Source: Cyborg Bionic Syst. 2026 May 7;7:0565. doi: 10.34133/cbsystems.0565 (PMC13150081; doi:10.34133/cbsystems.0565)

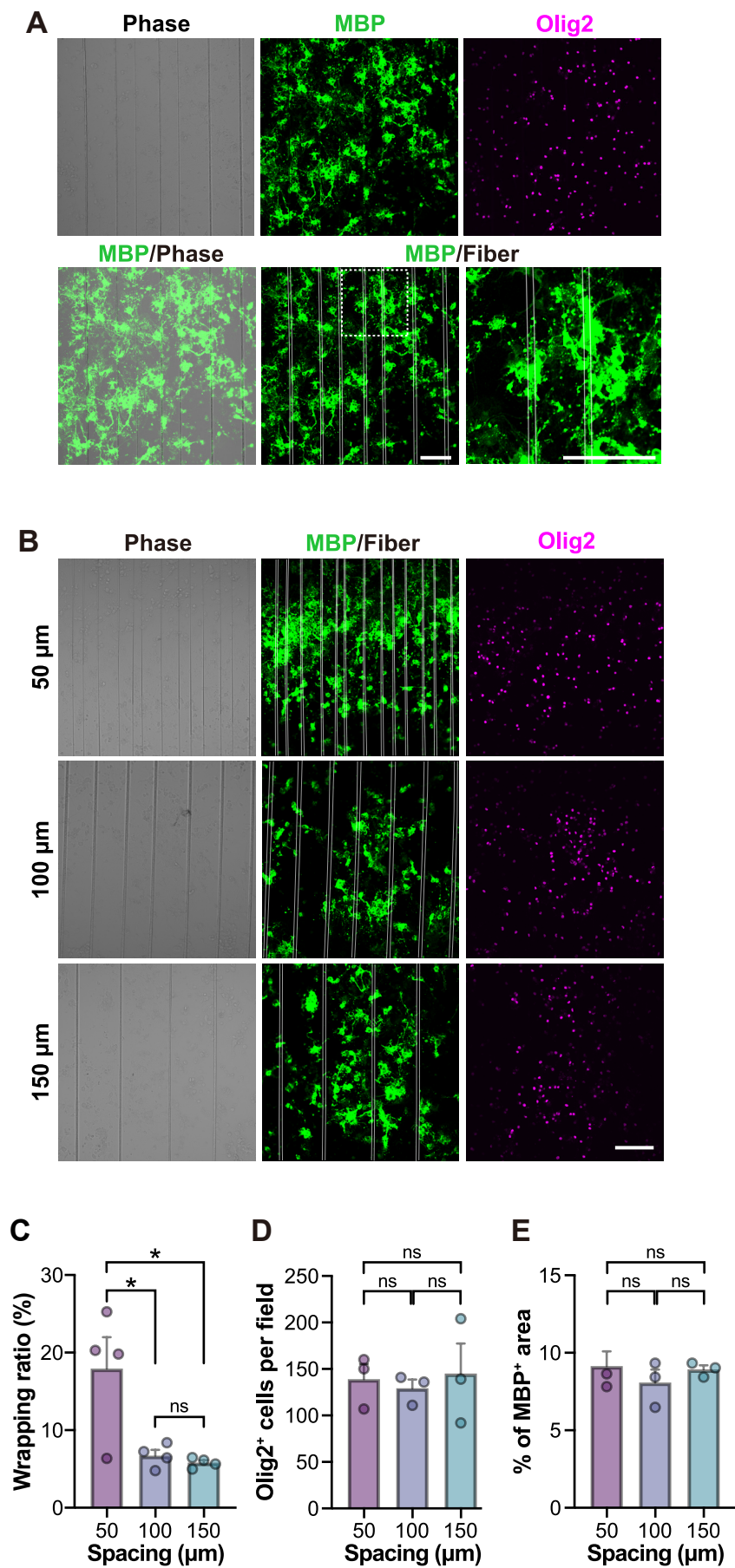

**Figure S1**

Supplement: Supplementary 1 — Figures S1 to S9 Table S1 [file cbsystems.0565.f1.zip › Quan et al FigS1.pdf]

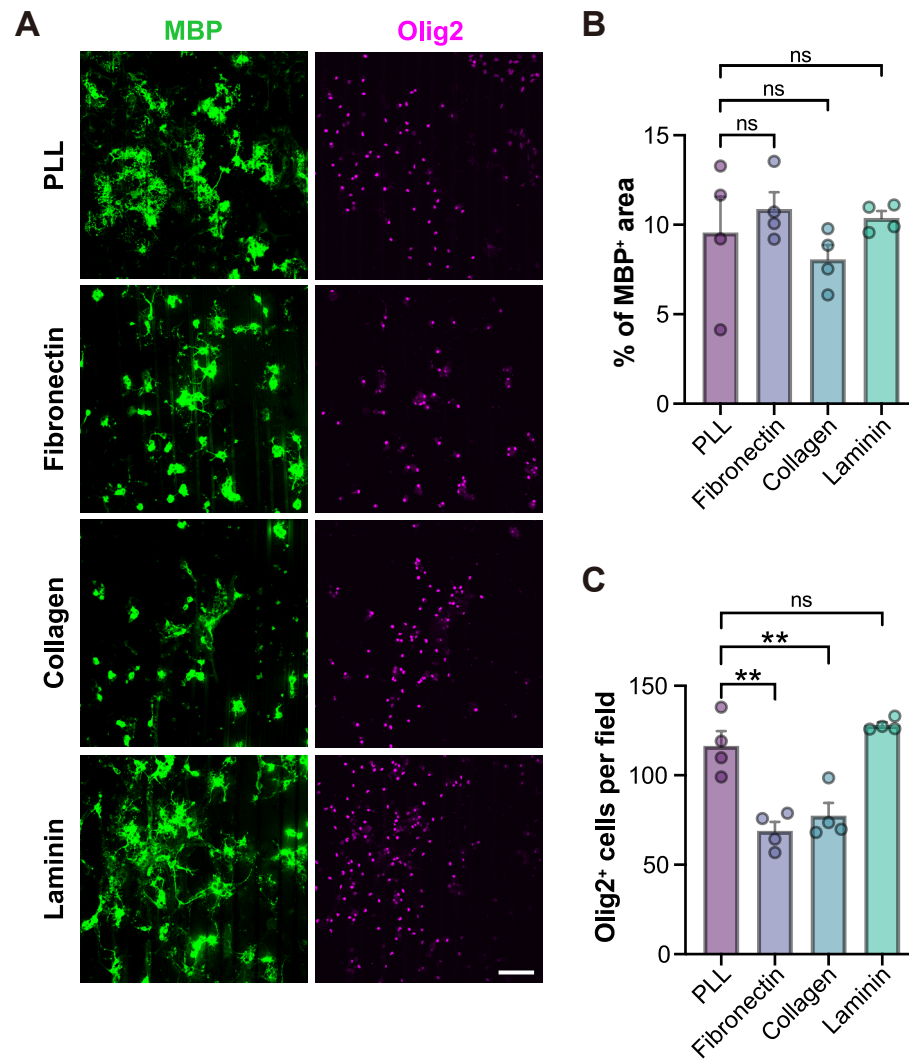

**Figure S2**

Supplement: Supplementary 1 — Figures S1 to S9 Table S1 [file cbsystems.0565.f1.zip › Quan et al FigS2.pdf]

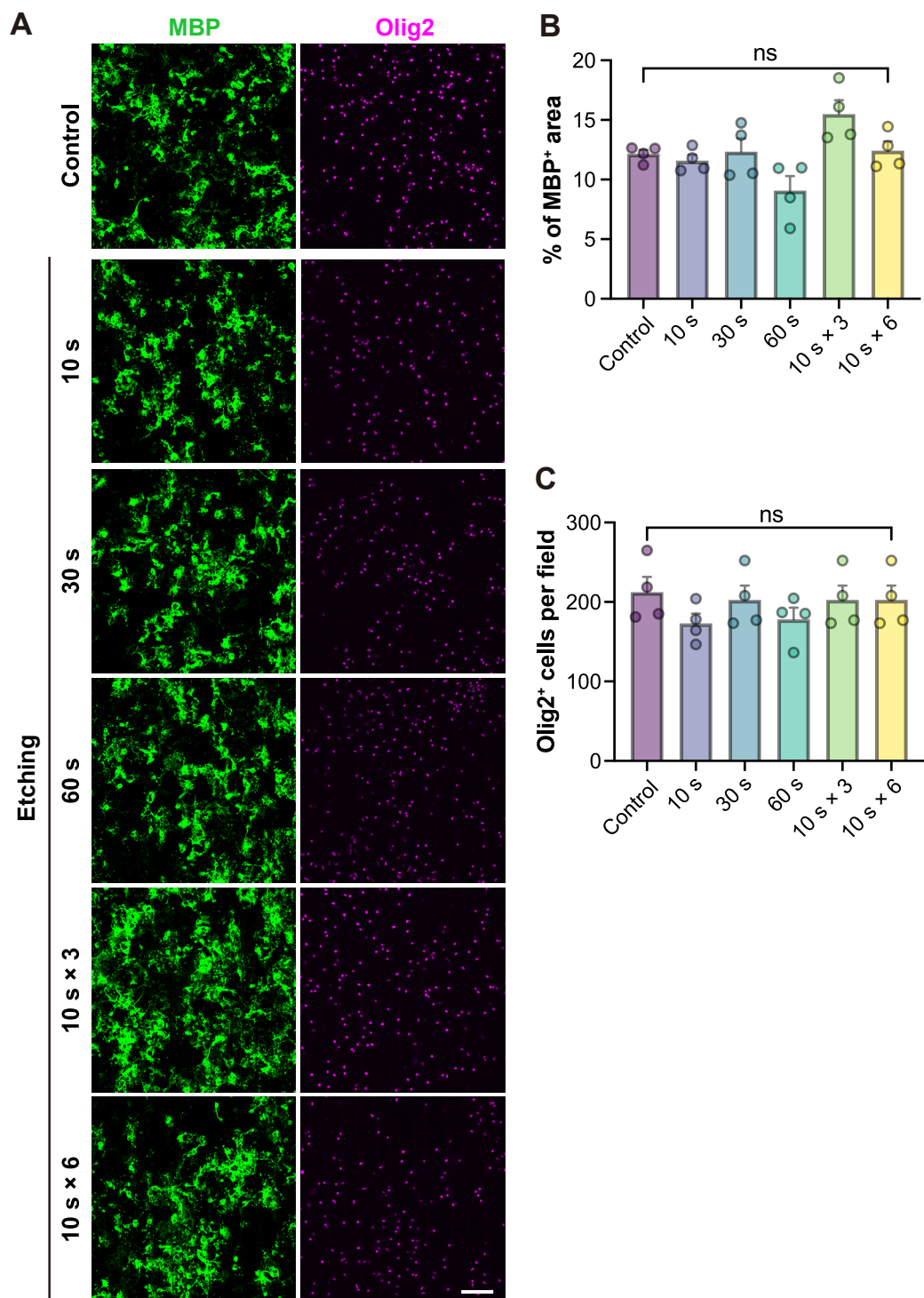

Figure S3

Supplement: Supplementary 1 — Figures S1 to S9 Table S1 [file cbsystems.0565.f1.zip › Quan et al FigS3.pdf]

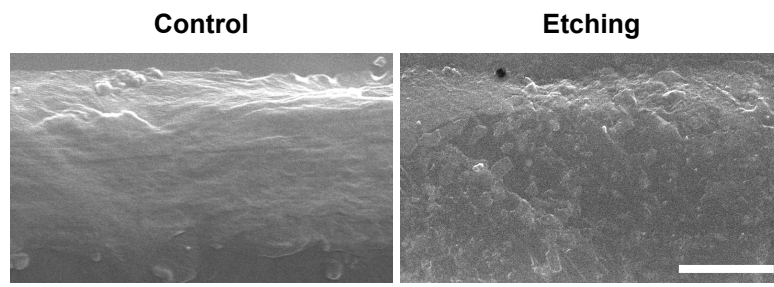

**Figure S4**

Supplement: Supplementary 1 — Figures S1 to S9 Table S1 [file cbsystems.0565.f1.zip › Quan et al FigS4.pdf]

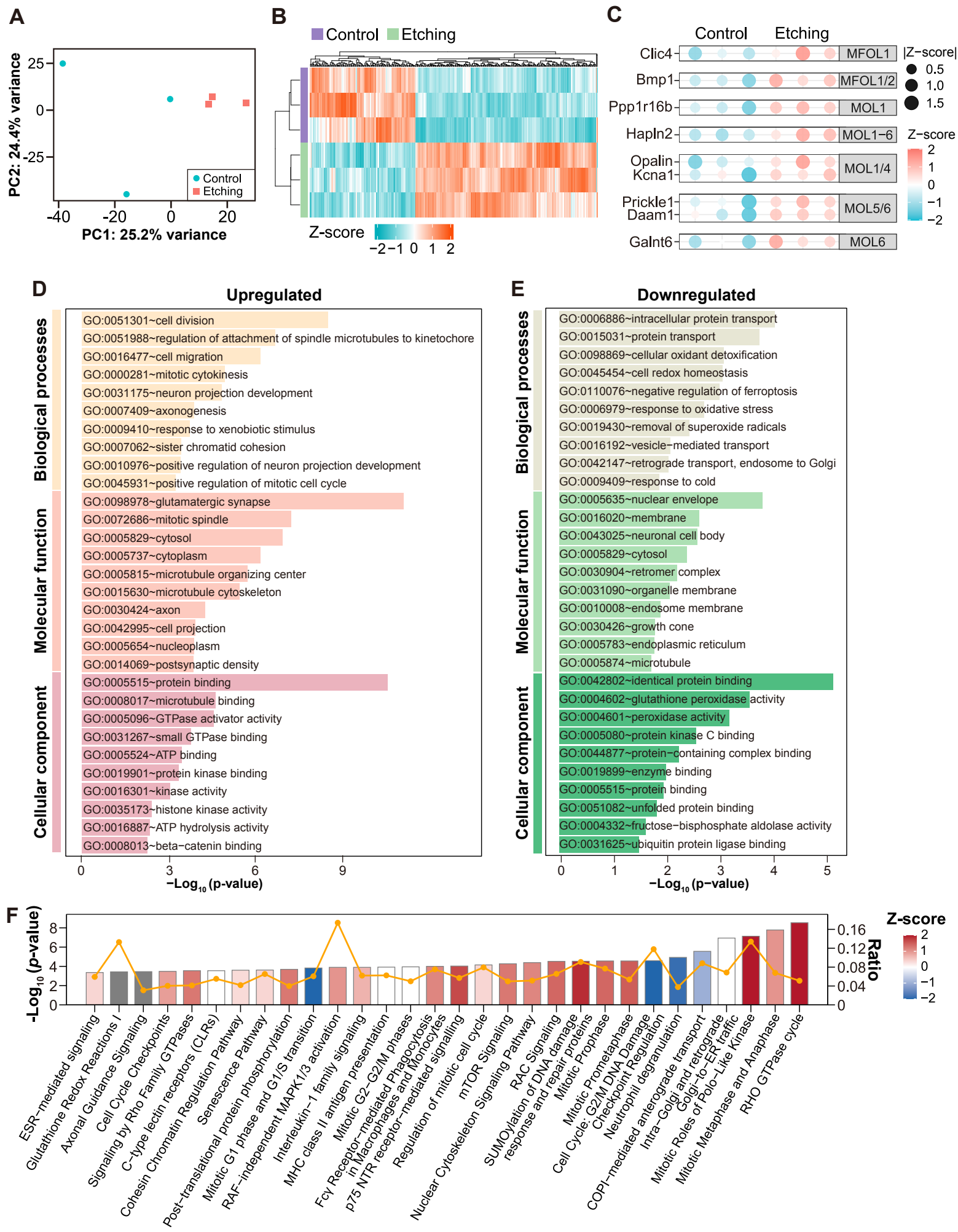

**Figure S5**

Supplement: Supplementary 1 — Figures S1 to S9 Table S1 [file cbsystems.0565.f1.zip › Quan et al FigS5.pdf]

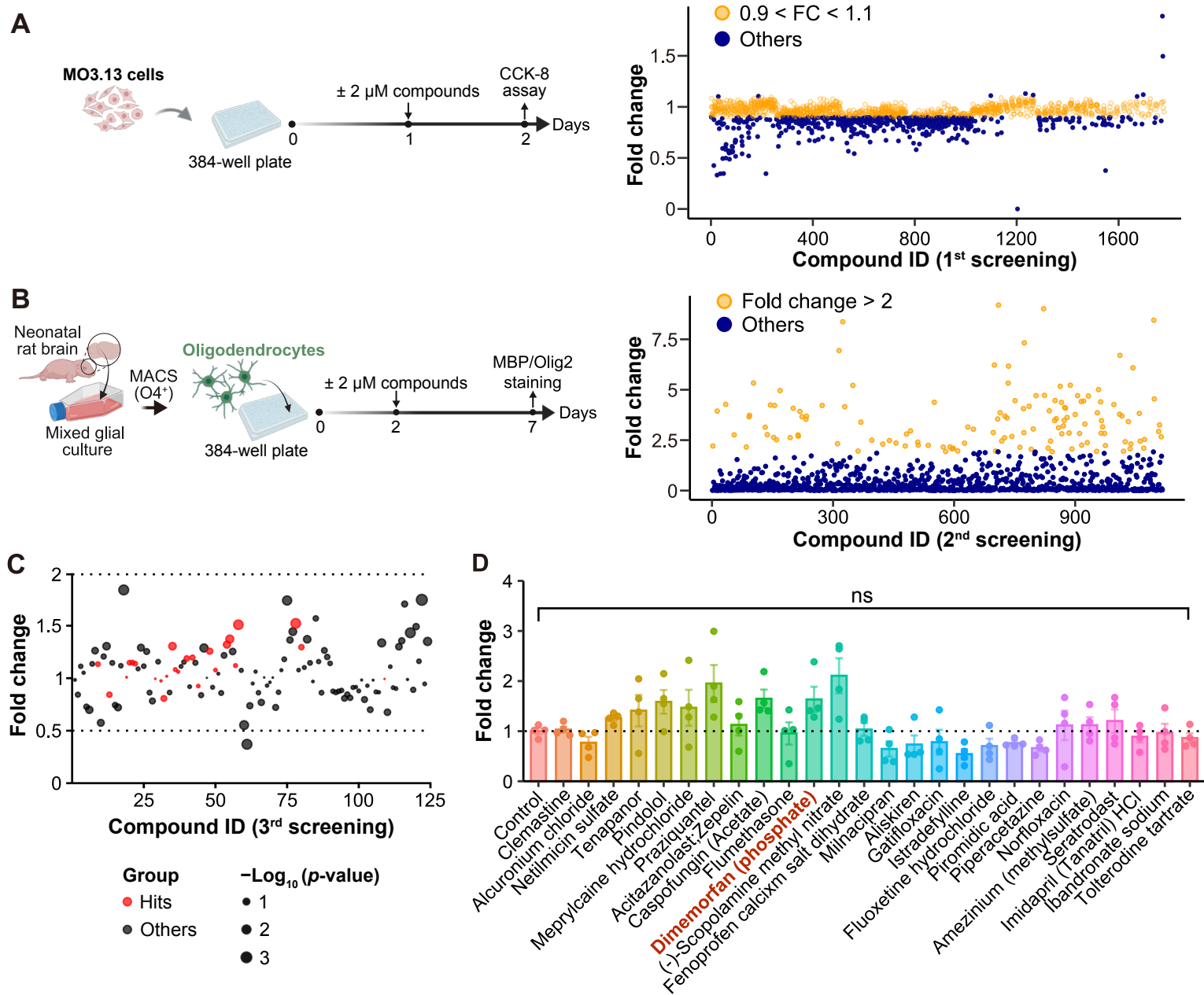

**Figure S6**

Supplement: Supplementary 1 — Figures S1 to S9 Table S1 [file cbsystems.0565.f1.zip › Quan et al FigS6.pdf]

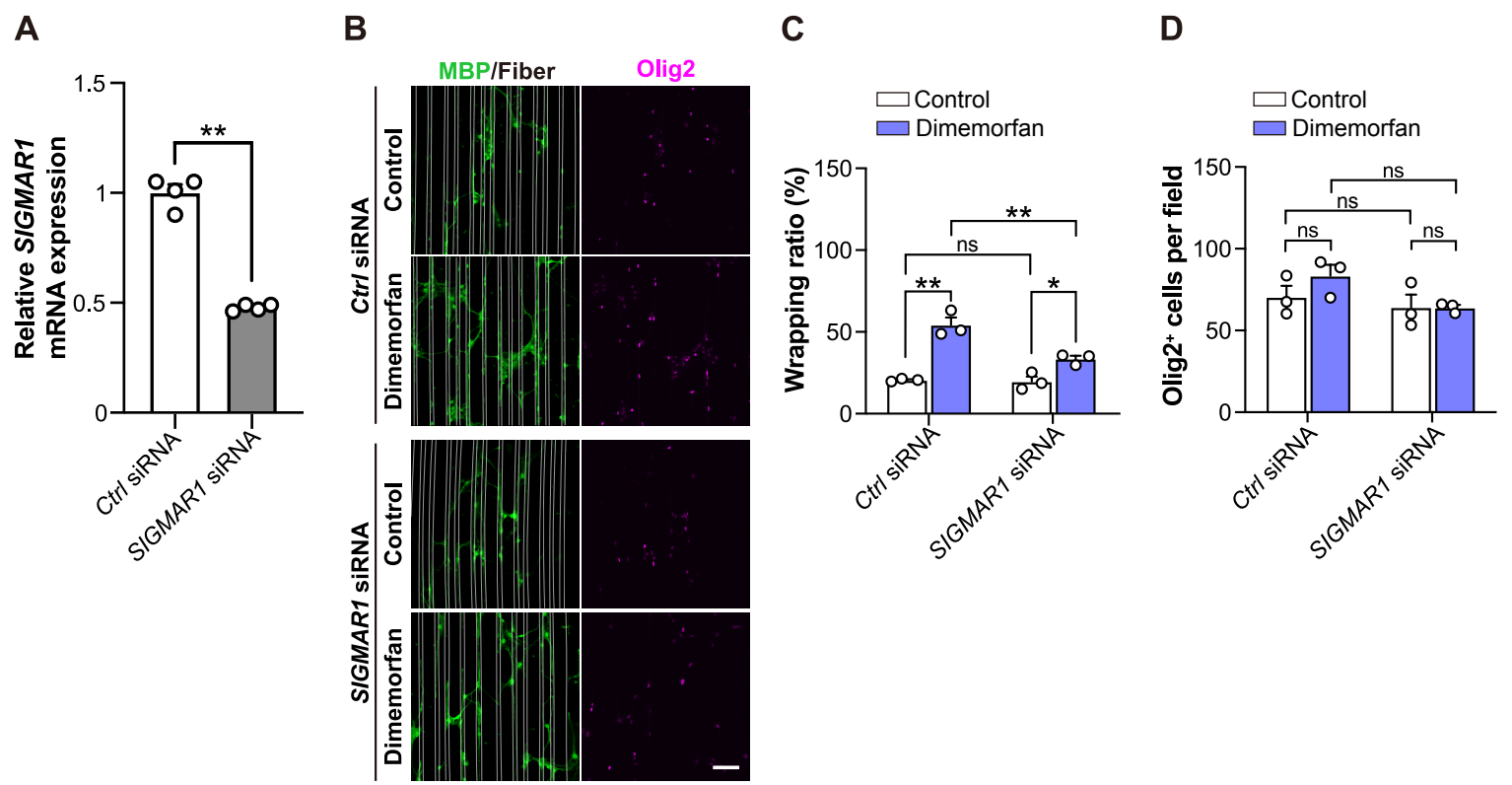

Figure S7

Supplement: Supplementary 1 — Figures S1 to S9 Table S1 [file cbsystems.0565.f1.zip › Quan et al FigS7.pdf]

A

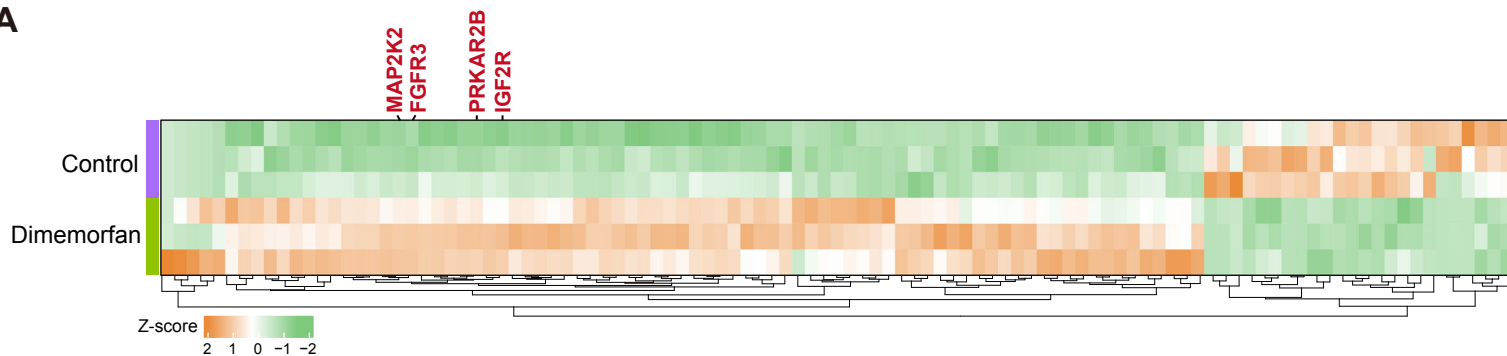

B

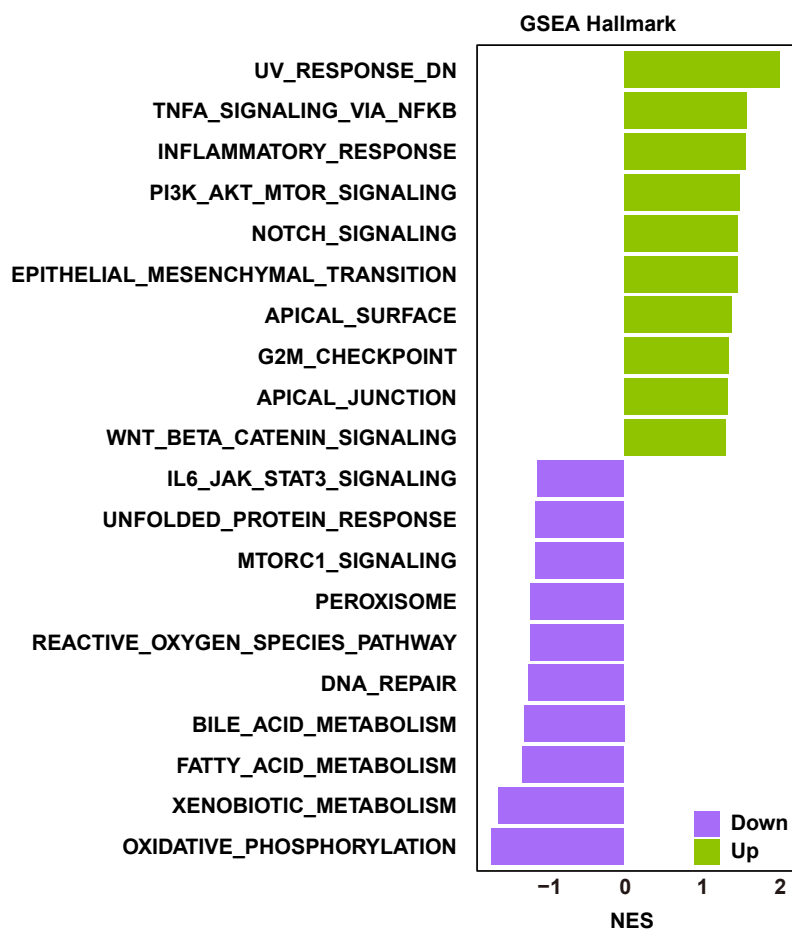

C

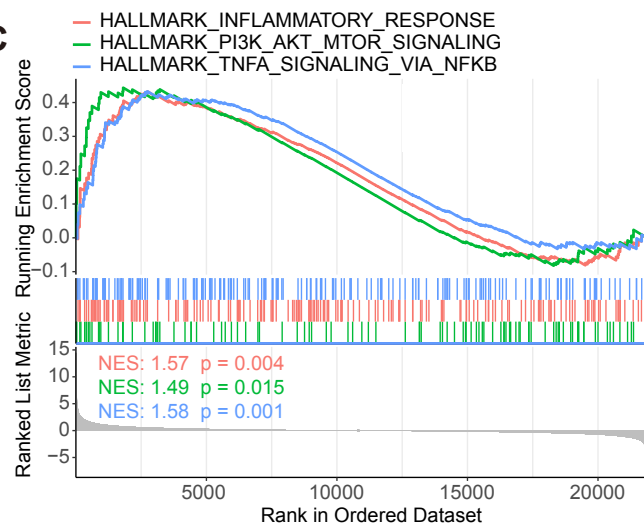

D

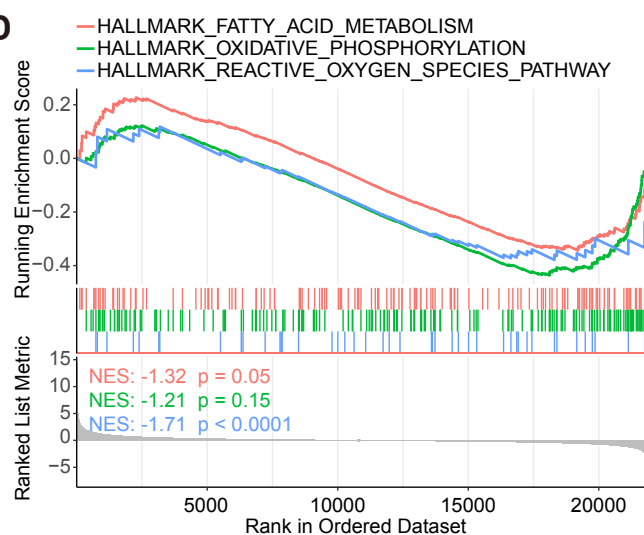

Figure S8

Supplement: Supplementary 1 — Figures S1 to S9 Table S1 [file cbsystems.0565.f1.zip › Quan et al FigS8.pdf]

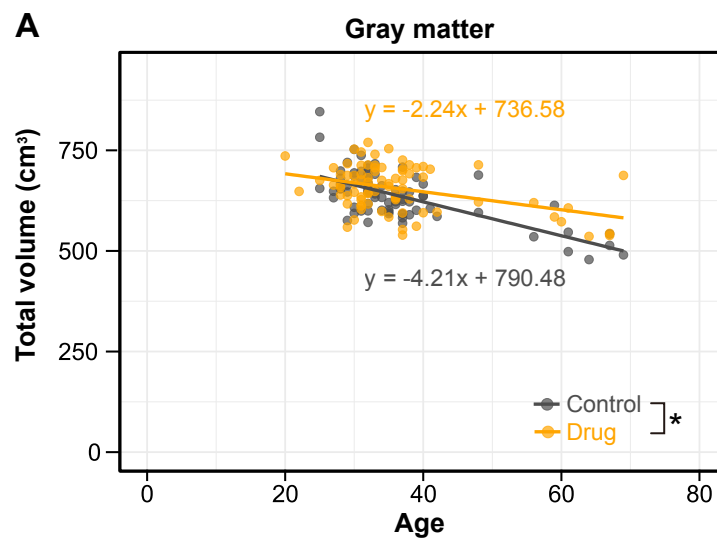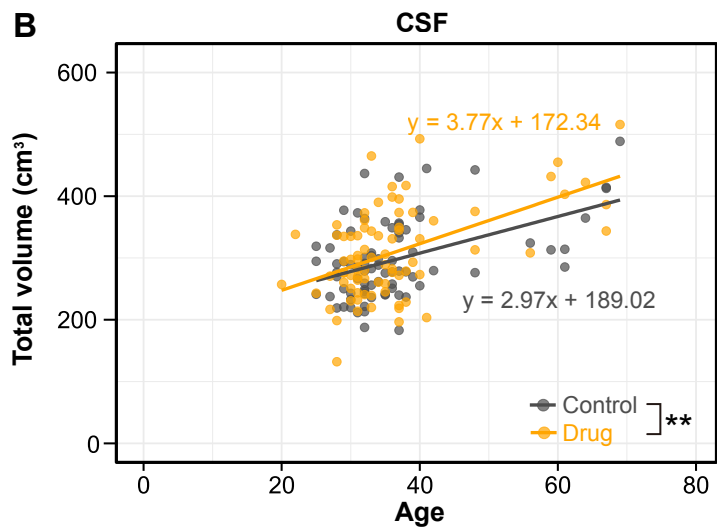

**Figure S9**

Supplement: Supplementary 1 — Figures S1 to S9 Table S1 [file cbsystems.0565.f1.zip › Quan et al FigS9.pdf]
